# Genomic insights into a novel species, *Dyella thailandensis* sp. nov., a cellulolytic and xylanolytic bacterium isolated from soil associated with leaf compost

**DOI:** 10.1038/s41598-025-33717-w

**Published:** 2026-01-09

**Authors:** Nahatai Intarapasit, Anon Thammasittirong, Sukanya Jeennor, Pattaraporn Yukphan, Sutticha Na-Ranong Thammasittirong

**Affiliations:** 1 Department of Science and Bioinnovation, Faculty of Liberal Arts and Science, Kasetsart University, Kamphaeng Saen Campus, Nakhon Pathom, 73140 Thailand; 2 Microbial Biotechnology Unit, Faculty of Liberal Arts and Science, Kasetsart University, Kamphaeng Saen Campus, Nakhon Pathom, 73140 Thailand; 3 Industrial Bioprocess Technology Research Team, Functional Ingredients and Food Innovation Research Group (IFIG), National Center for Genetic Engineering and Biotechnology (BIOTEC), National Science and Technology Development Agency (NSTDA), Pathum Thani, 12120 Thailand; 4 Microbial Diversity and Utilization Research Team, Thailand Bioresource Research Center (TBRC), National Center for Genetic Engineering and Biotechnology (BIOTEC), National Science and Technology Development Agency (NSTDA), Pathum Thani, 12120 Thailand

**Keywords:** *Dyella thailandensis*, Lignocellulose degradation, CAZyme, Polyphasic taxonomy, Genomics, Biotechnology, Microbiology, Molecular biology

## Abstract

The efficient enzymatic conversion of lignocellulose into fermentable sugars is essential for producing sustainable biofuels and biochemicals. During a screening for lignocellulose-degrading microorganisms, a novel bacterial strain, designated KULCS107^T^, was isolated from soil associated with leaf compost in Nakhon Pathom, Thailand. Phylogenetic analysis based on the 16S rRNA gene sequence placed the strain within the genus *Dyella*, with *Dyella ginsengisoli* Gsoil 3046^T^ as its closest described relative (99.0% sequence identity). However, genome-wide analyses revealed that strain KULCS107^T^ represents a distinct species. The digital DNA-DNA hybridization (dDDH) and average nucleotide identity (ANI) values between KULCS107^T^ and *D. ginsengisoli* Gsoil 3046^T^ were 36.2% and 88.9%, respectively—both substantially below the established thresholds for species delineation. The complete genome of strain KULCS107^T^ is 3.98 Mb, with a G + C content of 67.8%, and encodes 3,549 protein-coding genes, 48 tRNA genes, and 6 rRNA genes. Cellulase and xylanase production was confirmed when the strain was grown in carboxymethyl cellulose (CMC) and xylan broth, consistent with genomic predictions revealing numerous carbohydrate-active enzymes (CAZymes). Based on this polyphasic taxonomic evidence, strain KULCS107^T^ represents a novel species within the genus *Dyella*, for which the name *Dyella thailandensis* sp. nov. is proposed. The type strain is KULCS107^T^ (= TBRC 20486^T^ = KCTC 18276^T^ = NBRC 117363^T^).

**Supplementary Information:**

The online version contains supplementary material available at 10.1038/s41598-025-33717-w.

## Introduction

Lignocellulosic biomass is the most globally abundant renewable carbon source, primarily composed of cellulose, hemicellulose, and lignin. Its complex and recalcitrant structure poses a major challenge to efficient bioconversion. Conventional chemical and physical methods, such as acid or alkaline hydrolysis and steam explosion, have long been employed to deconstruct lignocellulose and enhance saccharification efficiency. However, these approaches often require harsh conditions, high energy input, and may generate inhibitory by-products^1,2^. In contrast, enzymatic hydrolysis provides a milder and environmentally friendly alternative, employing microorganisms and their enzymes to depolymerize lignocellulose into fermentable sugars, thereby supporting the sustainable production of biofuels and value-added biochemicals^3^. Although certain microorganisms possess the intrinsic ability to degrade native lignocellulosic materials, efficient bioconversion at laboratory or industrial scales generally requires pretreatment to improve substrate accessibility and enzymatic efficiency^2^. Consequently, the isolation and characterization of novel lignocellulose-degrading microorganisms are of great importance. The discovery of new species with these capabilities not only uncovers untapped genetic resources, advancing our understanding of microbial diversity in carbon cycling, but also provides promising and robust biocatalysts for future industrial applications.

The genus *Dyella* was first proposed in 2005, with *Dyella japonica* designated as the type species^4^. As of August 2025, the genus *Dyella* comprises 36 recognized species. This total, which includes 35 species with validly published names and one effectively published species (*Dyella sedimenti*) (https://lpsn.dsmz.de/genus/dyella), is consistent with the 36 reference genomes available in the NCBI database. Members of *Dyella* are ubiquitous in soil, having been isolated from diverse environments including forest^5,6^, cultivated^7,8^, and grassland soils^9^, as well as specialized habitats such as cliff soils^10^. However, some *Dyella* strains have been recovered from non-soil environments, including rock surfaces^11^ and winery sediment^12^. Phenotypically, species in this genus are characterized typically as Gram-stain-negative and aerobic. Cells are rod-shaped and colonies are yellow-pigmented. The major respiratory quinone is ubiquinone-8^5,12^. Several *Dyella* strains have demonstrated diverse metabolic capabilities relevant to both environmental and applied microbiology, including tolerance to activated aluminum ions (Al³⁺) and inhibition of the fungal phytopathogen *Fusarium oxysporum* f. sp. *melonis*^7^, and degradation of pollutants such as biphenyl^13^ and triclosan^14^. In addition, some strains produce the food processing enzyme isoamylase^15^ and have been investigated as biofertilizers^16^. While these functions underscore their potential, the capacity for efficient plant biomass degradation among *Dyella* species remains less explored.

In the current study, a Gram-negative bacterium, designated strain KULCS107^T^, was isolated from soil associated with decomposing leaf litter during a screening for lignocellulolytic microorganisms. This isolate exhibited the largest combined hydrolysis zones for both cellulase and xylanase activities, indicating its strong potential for lignocellulose degradation and warranting further investigation. Accordingly, the aims of the current study were to determine the taxonomic position of strain KULCS107^T^ through a polyphasic approach and to perform whole-genome sequencing and analysis, with a particular emphasis on identifying its carbohydrate-active enzymes (CAZymes), to assess both its novelty within the genus *Dyella* and its potential for biotechnological applications.

## Materials and methods

### Bacterial isolation and screening

A soil sample associated with leaf compost was collected from a residential garden in Kamphaeng Saen, Nakhon Pathom, Thailand (13°57′34.8″ N, 100°00′07.9″ E) in June 2023. The sample was enriched in carboxymethyl cellulose (CMC) broth (5 g/L CMC, 0.5 g/L MgSO₄·7 H₂O, 0.5 g/L yeast extract, 1 g/L KH₂PO₄, 1 g/L (NH_4_)_2_SO_4_, and 1.0 g/L KCl) at 37 °C with shaking at 150 rpm for 1–2 days. Then, the enriched culture was serially diluted, spread onto CMC agar, followed by incubation at 37 °C for 1–2 days to obtain bacterial colonies. Following purification, the isolates were screened for cellulolytic and xylanolytic on CMC and xylan agar, respectively, using the Gram’s iodine overlay method^17^. A clear hydrolysis zone, or halo, around a colony indicated positive activity. Pure cultures were preserved in 20% (w/v) glycerol at -80 °C.

### 16S rRNA gene and phylogenetic analysis

Genomic DNA was extracted using a MagAttract HMW DNA kit (Qiagen, Hilden, Germany), following the manufacturer’s instructions. The 16S rRNA gene was amplified using PCR based on the universal bacterial primers 27F and 1492R^18^, with the amplicon being purified and sequenced by Bionics Inc. (Seoul, Republic of Korea). The partial 16S rRNA sequence (1,459 bp) was deposited in GenBank under accession number PV635141. For the phylogenetic analysis, the full-length 16S rRNA gene sequence of strain KULCS107^T^ (1,545 bp) was extracted from the whole-genome sequence using the ContEst16S tool^19^. The sequence was initially compared against the EzBioCloud 16S rRNA gene database^20^ to determine the most closely related taxa. For the phylogenetic tree construction, complete 16S rRNA gene of the type strains for all 36 recognized *Dyella* species and all 7 type strains of the genus *Frateuria* were retrieved from their respective genome assemblies available in the NCBI database. Multiple sequence alignment was performed using MUSCLE^21^. The phylogenetic trees were constructed using the MEGA11^22^ software based on the maximum-likelihood^23^, neighbor-joining^24^, and minimum-evolution^25^ methods. Evolution distances were calculated using a Kimura two-parameter model^26^ and bootstrap values were calculated based on 1,000 replications.

### Whole-genome sequencing and analysis

Genomic DNA was sequenced using a hybrid approach combining PacBio Sequel II long-reads and Illumina HiSeq short-reads (Macrogen, Seoul, Republic of Korea). A *de novo* hybrid assembly was generated by first assembling the long reads into a consensus sequence using Trycycler v.0.5.0^27^. This consensus was then polished with the Illumina short reads using Polypolish v.0.5.0^28^. The quality and contiguity of the final genome assembly were evaluated using QUAST^29^, while genome completeness and contamination were assessed using CheckM v1.2.3^30^. The complete genome was annotated using the NCBI Prokaryotic Genome Annotation Pipeline (PGAP)^31^ and was deposited in GenBank under accession number CP192735.

The functional potential of the genome was characterized using multiple tools. A comprehensive functional annotation was performed using the Rapid Annotation using Subsystem Technology (RAST) server^32^. Genes were classified further into Clusters of Orthologous Groups (COG) using eggNOG-mapper^33^, while the CAZymes were predicted with the dbCAN3 meta v2.2.12^34^. A circular map of the genome was generated using Proksee^35^.

### Phylogenomics and comparative genomics

Phylogenomic analysis was performed using the Type Strain Genome Server (TYGS)^36^. A phylogenomic tree of strain KULCS107^T^ and all 36 type strains of the genus *Dyella* and all 7 type strains of the genus *Frateuria* was constructed via the Bacterial and Viral Bioinformatics Resource Center (BV-BRC)^37^. This tree was based on a concatenated alignment of 1,000 conserved single-copy orthologous automatically selected by the BV-BRC Phylogenetic Analysis tool, with branch support values calculated using 100 rapid bootstrap replicates in RAxML^38^. A heatmap and dendrogram of pairwise ANI values between strain KULCS107^T^ and all type strains of the genera *Dyella* and *Frateuria* were constructed using the Integrated Prokaryotes Genome and Pan-genome Analysis (IPGA) web server^39^. The average nucleotide identity (ANI) and digital DNA-DNA hybridization (dDDH) of related type strains (*Dyella ginsengisoli* Gsoil 3046^T^, *Dyella thiooxydans* ATSB10^T^, *Dyella aluminiiresistens* A6^T^, *Dyella soli* JS12-10^T^, and *Dyella lutea* Sa^T^) were calculated using the OrthoANIu algorithm^40^ and the Genome-to-Genome Distance Calculator (GGDC 3.0, formula 2)^41^, respectively. Orthologous gene clusters between strain KULCS107^T^ and its closest relatives (*D. ginsengisoli* Gsoil 3046^T^, *D. thiooxydans* ATSB10^T^, and *D. lutea* SaT) were identified using the OrthoVenn3 server^42^.

### Morphological and physiological characterization

Colony morphology was examined on R2A agar after incubation at 35 °C for 2 days. The Gram reaction was determined using the non-staining KOH method^43^ and a standard staining protocol^44^. Cell morphology and Gram reaction were observed under a light microscope (CX31, Olympus; Tokyo, Japan) at ×1000 magnification. Motility was assessed using the hanging drop technique. For determination of cell size and flagellation, cells were negatively stained with 1% (w/v) uranyl acetate and examined using transmission electron microscopy (HT7700, Hitachi; Tokyo, Japan) at 80 kV. Catalase activity was tested based on bubble formation in 3% (v/v) hydrogen peroxide, while oxidase activity was determined using 1% (w/v) tetramethyl-*p*-phenylenediamine. The temperature range for growth was determined on R2A agar incubated at 10, 15, 20, 25, 30, 35, 37, 40, and 45 °C for 2 days. The pH range for growth (pH 4.0–10.0, at 0.5-unit intervals) was evaluated in R2A broth at 35 °C for 5 days using 50 mM buffer systems: sodium citrate (pH 4.0–5.5), phosphate (pH 6.0–8.0), and sodium borate (pH 8.5–10.0). Salt tolerance was tested in R2A broth supplemented with NaCl (0–5% w/v, at 0.5% intervals) under the same conditions. Optimal temperature for growth was determined based on the best growth observed on R2A agar for 2 days. The optima for pH and NaCl concentration were determined from the highest optical density in R2A broth, with all incubations lasting for 5 days.

Growth on different media, including R2A agar, Luria-Bertani (LB) agar, nutrient agar (NA), and MacConkey agar was tested and observed after 2 days incubation at 35 °C. Anaerobic growth was examined on R2A agar incubated in a GasPak anaerobic jar (BBL GasPak™, Becton, Dickinson; Sparks, MD, USA) at 35 °C for 14 days. Enzymatic and biochemical characteristics were determined using API ZYM and API 20NE test kits (bioMérieux, Marcy-l’Étoile, France) according to the manufacturer’s instructions. Positive and negative results were determined based on distinct color changes or growth as specified in the reading table, following the manufacturer’s interpretation guidelines, whereas a weakly positive result (w) referred to an ambiguous or faint color reaction, or weak growth, that did not fully correspond to the defined positive or negative descriptions. All tests were carried out in triplicate.

### Chemotaxonomic characterization

Cells grown in R2A broth at 35 °C for 3 days were harvested for chemotaxonomic analyses of respiratory quinones, fatty acid profiles, and polar lipid compositions. The respiratory quinones were extracted according to the method of Yamamoto^45^, purified using thin-layer chromatography (silica gel; diethyl ether: hexane, 3:1, v/v), and analyzed using high-performance liquid chromatography (HPLC) as described by Komagata and Suzuki^46^. Cellular fatty acids were analyzed using the Sherlock Microbial Identification System^47^ with identification via the TSBA6 database (version 6.2B). Total lipids were extracted from freeze-dried cells using a modification of the method by Folch et al.^48^, and the total lipid content was determined gravimetrically, expressed as milligrams per gram of dry cell weight (mg/g dry cell weight). Polar lipid classes within this extract were then quantified using HPLC with a charged aerosol detector (Corona, ESA Biosciences; Chelmsford, MA, USA) following Khoomrung et al.^49.^ Individual lipids were identified by comparing retention times with authentic standards (Sigma-Aldrich, St. Louis, Mo, USA) and quantified using calibration curves (R² > 0.95). For one-dimensional HP-TLC confirmation, lipid extracts in chloroform: methanol (2:1, v/v) were applied to a silica gel plate (60 F₂₅₄, CAMAG; Muttenz, Switzerland). The plate was developed in a chloroform-methanol-glacial acetic acid (65:25:10, v/v/v) solvent system^50^. Separated lipid classes were visualized with iodine vapor^51^ and identified by comparing R_ƒ_ values with authentic standards. Additionally, polar lipids were extracted and identified using two-dimensional TLC according to the method of Minnikin et al.^52^.

### Crude enzyme production and enzymatic assay

The strain KULCS107^T^ was cultured in 50 mL of basal medium^17^ supplemented with either 10.0 g/L CMC or 10.0 g/L xylan (pH 7.0). Cultures were incubated at 35 °C for 2 days with shaking at 150 rpm. After incubation, the culture was centrifuged (10,000 rpm, 15 min, 4 °C), and the cell-free supernatant was collected as the crude enzyme source for activity assays.

Cellulase (CMCase) and xylanase activities were quantified by measuring the release of reducing sugars using the 3,5-dinitrosalicylic acid (DNS) method. The CMCase activity assay, based on Namnuch et al.^53^, consisted of 0.5 mL of diluted crude enzyme and 0.5 mL of 1% (w/v) CMC in  50 mM sodium citrate buffer (pH 4.8), incubated at 50 °C for 30 min. The commercial cellulase, Celluclast^®^ 1.5 L, (Sigma-Aldrich, St. Louis, MO, USA) was used for positive control. The xylanase activity assay, modified from Bailey et al.^54^, contained 0.2 mL of crude enzyme and 1.8 mL of 1% (w/v) xylan in 50 mM Tris-HCl buffer (pH 7.0), incubated at 50 °C for 5 min. The commercial xylanase, X2753, (Sigma-Aldrich, St. Louis, MO, USA) was used for positive control. For both assays, the reactions were terminated by adding 3.0 mL of DNS reagent, followed by boiling for 5 min. After cooling, the absorbance was measured at 540 nm. The amount of released reducing sugar was quantified using a glucose standard curve for CMCase and a xylose standard curve for xylanase. Negative controls for each assay consisted of enzyme without substrate and substrate without enzyme. All enzymatic assays were performed in triplicates. One unit (U) of activity was defined as the amount of enzyme required to release 1 µmol of the respective monosaccharide (glucose or xylose) per minute under the specified assay conditions.

## Results and discussion

### Isolation and screening of a novel bacterial strain

The screening for lignocellulolytic microorganisms from soil associated with decomposing leaf litter, termite mound soil, and cow dung yielded 92 isolates capable of utilizing CMC. Subsequent plate assays identified 23 isolates with cellulolytic and 16 isolates with xylanolytic activity, based on the formation of hydrolysis zones on CMC and xylan agar, respectively. Of these 39 isolates, only 7 demonstrated both activities. The isolate designated KULCS107^T^, which exhibited the highest combined cellulase and xylanase activity as indicated by the largest hydrolysis zones (Fig. S1), was selected for further study.

### 16S rRNA gene and phylogenetic analysis

The nearly complete 16S rRNA gene sequence of strain KULCS107^T^ was 1,459 bp in length and was deposited in GenBank under accession number PV635141. A comparative analysis using the EzBioCloud database indicated that strain KULCS107^T^ shared the highest 16S rRNA gene sequence identity with *Dyella ginsengisoli* Gsoil 3046^T^ (99.0%). Sequence identities with other related type strains were 98.6% to *Dyella thiooxydans* ATSB10^T^, 98.1% to *Dyella aluminiiresistens* A6^T^, 98.0% to *Dyella soli* JS12-10^T^, and 97.8% to *Dyella lutea* Sa^T^ (Table 1).

**Table 1:** Comparison of 16S rRNA gene sequence identity, ANI, and dDDH values between strain KULCS107^T^ and its closest relatives.

Species	Strain	16 S rRNA gene identity (%)	ANI value (%)	dDDH value (%)
*D. ginsengisoli*	Gsoil 3046^T^	99.0	88.9	36.2
*D. lutea*	Sa^T^	97.8	84.3	27.4
*D. thiooxydans*	ATSB10^T^	98.6	84.1	26.9
*D. soli*	JS12-10^T^	98.0	77.9	21.5
*D. aluminiiresistens*	A6^T^	98.1	77.7	21.5

**Fig. 1:**
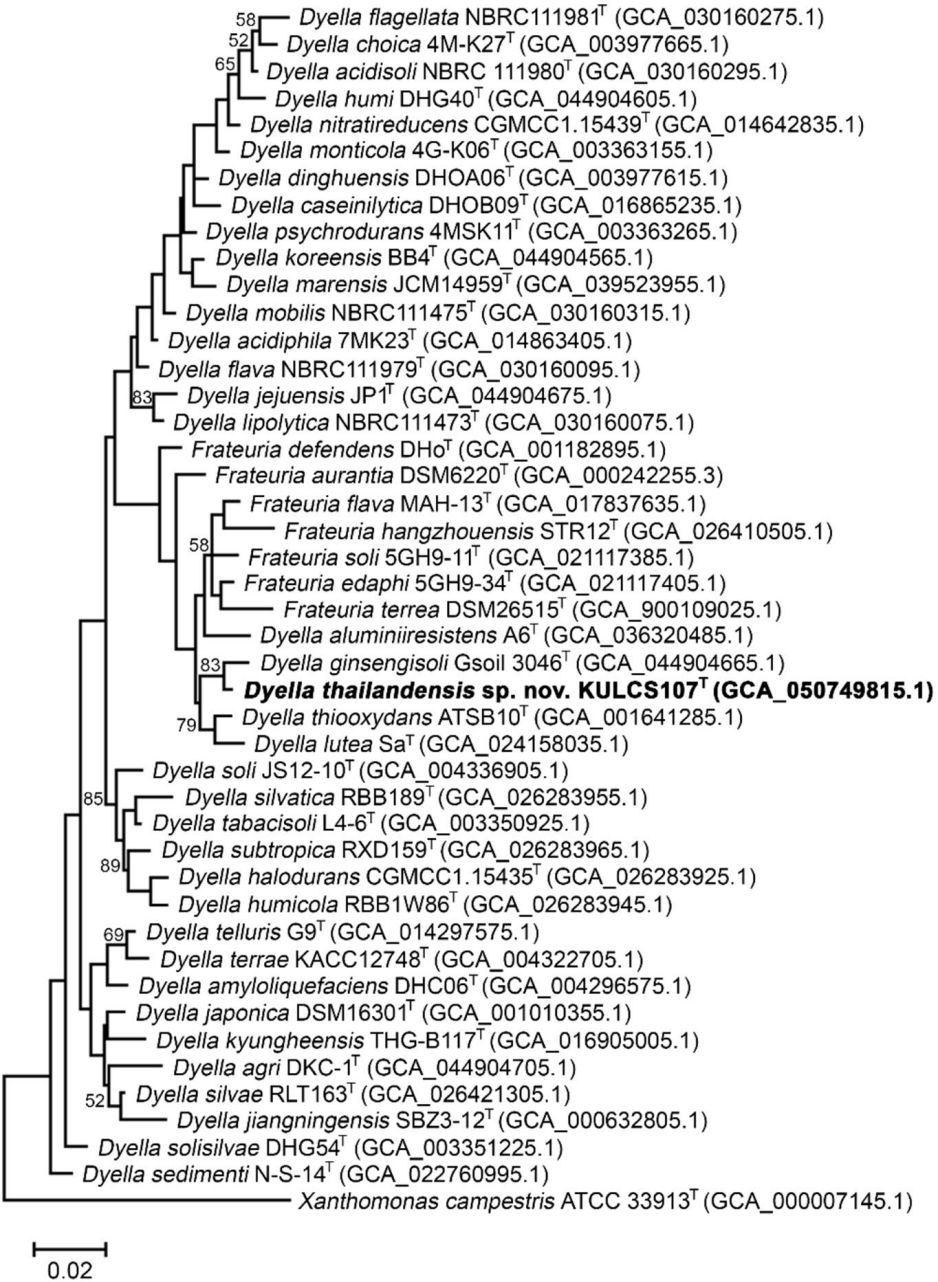
Maximum-likelihood phylogenetic tree based on 16S rRNA gene sequences illustrating position of strain KULCS107^T^ relative to all closely related species of the genera *Dyella* and *Frateuria*, with *Xanthomonas campestris* ATCC 33913^T^ as the outgroup. Bootstrap values (based on 1,000 replications) greater than 50% are shown at the nodes. The analysis was based on an alignment of 1,545 nucleotide positions. GenBank accession numbers are in parentheses. Bar, 0.02 substitutions per nucleotide site.

Phylogenetic analyses based on complete 16S rRNA gene sequences of all 36 recognized *Dyella* type strains and all 7 type strains of the genus *Frateuria*, using the maximum-likelihood (Fig. 1), neighbor-joining (Fig. S2), and minimum-evolution (Fig. S3) algorithms, consistently positioned strain KULCS107^T^ within the genus *Dyella*. In the resulting trees, the strain formed a distinct phylogenetic lineage with its closest relative, *D. ginsengisoli* Gsoil 3046^T^. Since the 16S rRNA gene sequence identity between these 2 strains exceeds the 98.65% threshold commonly used for species delineation^55^, further genome-based analyses were performed to clarify the taxonomic status of strain KULCS107^T^.

### Genomic features, phylogenomic and genomic analysis

A hybrid genome assembly using both PacBio long reads (328x coverage) and Illumina short reads (901x coverage) resulted in a complete, circular chromosome for strain KULCS107^T^, with no plasmid detected. Assessment using the CheckM v1.2.3 confirmed the high quality of the assembly, indicating 99.62% completeness with 0.57% contamination. A summary of the key genomic features and a comparison with closely related species are presented in Table 2. The genome has a size of 3,978,691 bp, a G + C content of 67.8%, and contains 3,549 coding sequences (CDS), 6 rRNA genes (2 5S, 2 16S, 2 23S) and 48 tRNA genes. The two 16S rRNA gene copies were identical and showed 100% identity with the amplified 16S rRNA gene sequence. Notably, as shown in Table 2, the genome of KULCS107^T^ is smaller and contains fewer CDS than its relatives, with the exception of *D. aluminiresistens* A6^T^.

**Table 2:** Comparison of genomic features between strain KULCS107^T^ and its closest relatives. Strains: 1, KULCS107^T^; 2, *D. ginsengisoli* Gsoil 3046^T^; 3, *D. lutea* Sa^T^; 4, *D. thiooxydans* ATSB10^T^; 5, *D. soli* JS12-10^T^; 6, *D. aluminiiresistens* A6^T^. Data for strains 2–6 were retrieved from GenBank.

Genomic feature	1	2	3	4	5	6
Genome size (Mb)	3.98	4.26	4.40	4.23	8.19	3.72
Contig	1	19	8	1	19	1
GC content (%)	67.8	67.5	67.9	67.9	65.0	65.0
CDS	3,549	3,822	3,987	3,782	7,218	3,239
rRNA genes	6	5	6	6	13	6
tRNA genes	48	46	50	49	96	50

Genome-based taxonomic analysis using the Type Strain Genome Server (TYGS) placed strain KULCS107^T^ within the genus *Dyella*, forming a distinct phylogenomic lineage most closely related to *D. ginsengisoli* Gsoil 3046^T^ (Fig. S4). This relationship was further quantified using pairwise genomic comparisons, which yielded ANI and dDDH values of 88.9% and 36.2%, respectively, when compared with *D. ginsengisoli* Gsoil 3046^T^ (Table 1). Both values fall well below the established species delineation thresholds of 95% for ANI and 70% for dDDH^56^, confirming that strain KULCS107^T^ represents a novel species within the genus *Dyella.*

**Fig. 2:**
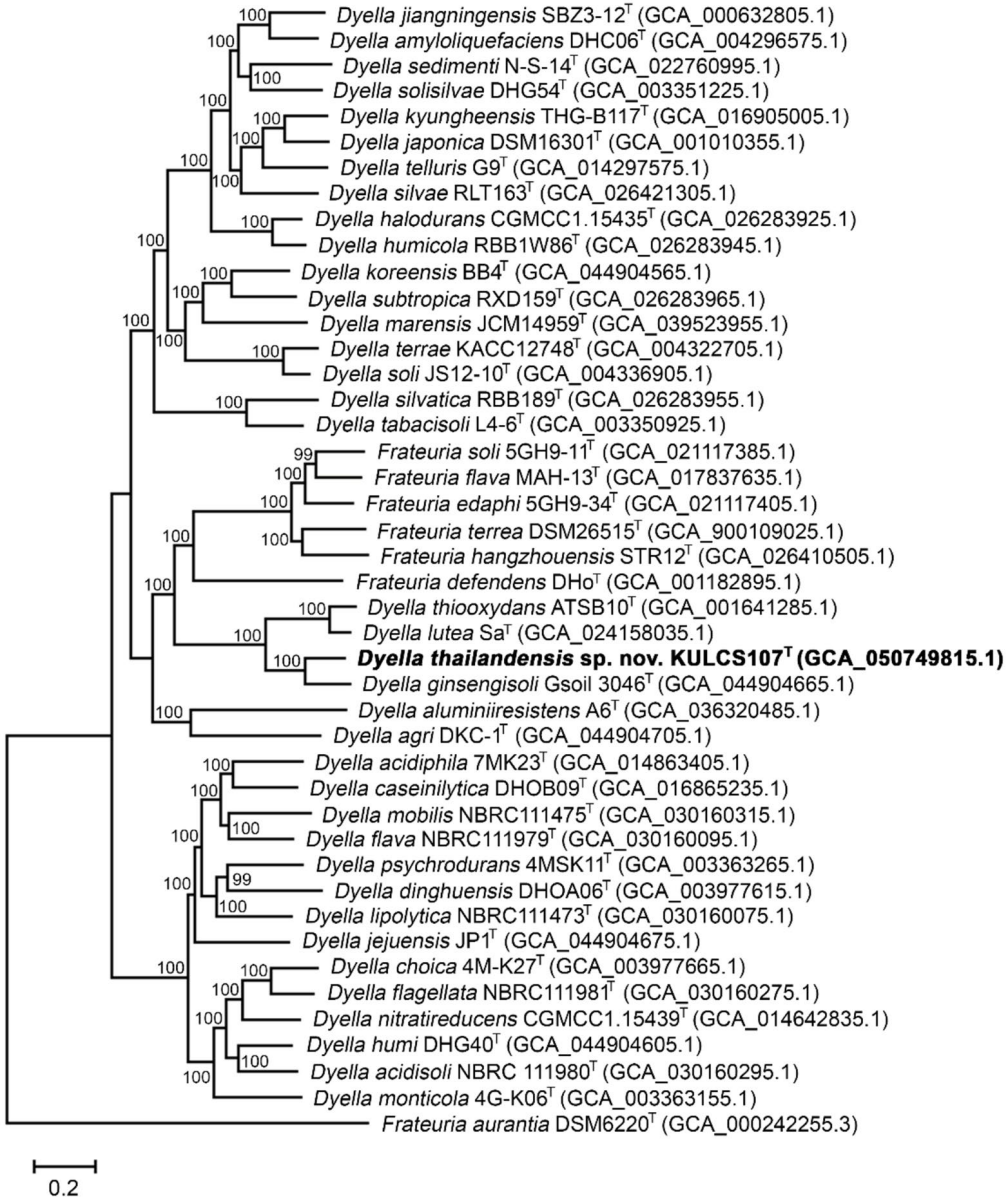
Phylogenomic tree constructed using 1,000 conserved single-copy orthologous genes, illustrating the phylogenetic relationship of strain KULCS107^T^ with all type strains of the genera *Dyella* and *Frateuria.* Bootstrap support values based on 100 replications are shown at the nodes. GenBank accession numbers are given in parentheses. Bar, 0.2 substitutions per nucleotide site.

**Fig. 3:**
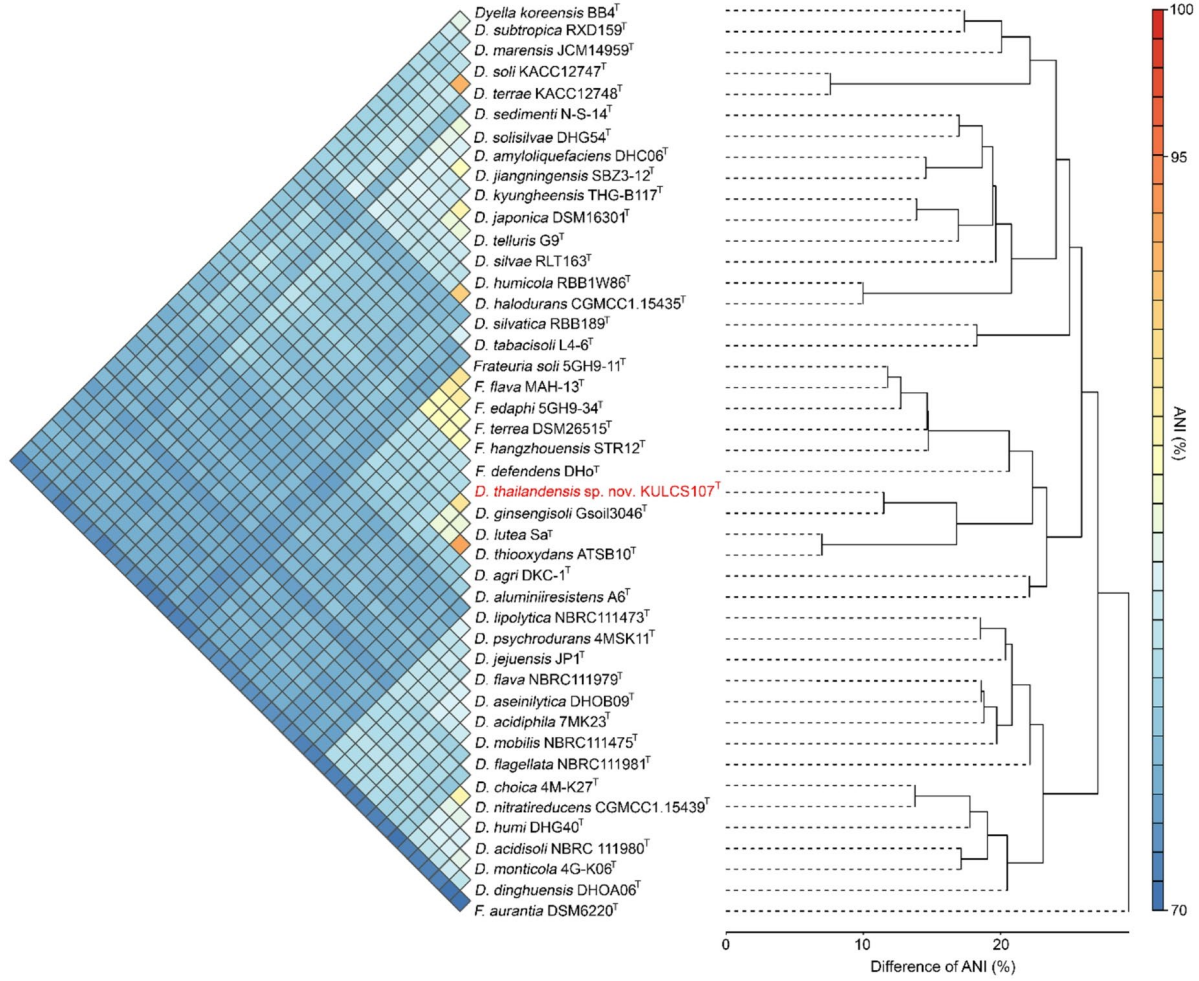
Heatmap and dendrogram of pairwise ANI values between strain KULCS107^T^ and all type strains of the genera *Dyella* and *Frateuria*. Color gradient represents ANI values from 70% (blue) to 100% (red).

**Fig. 4:**
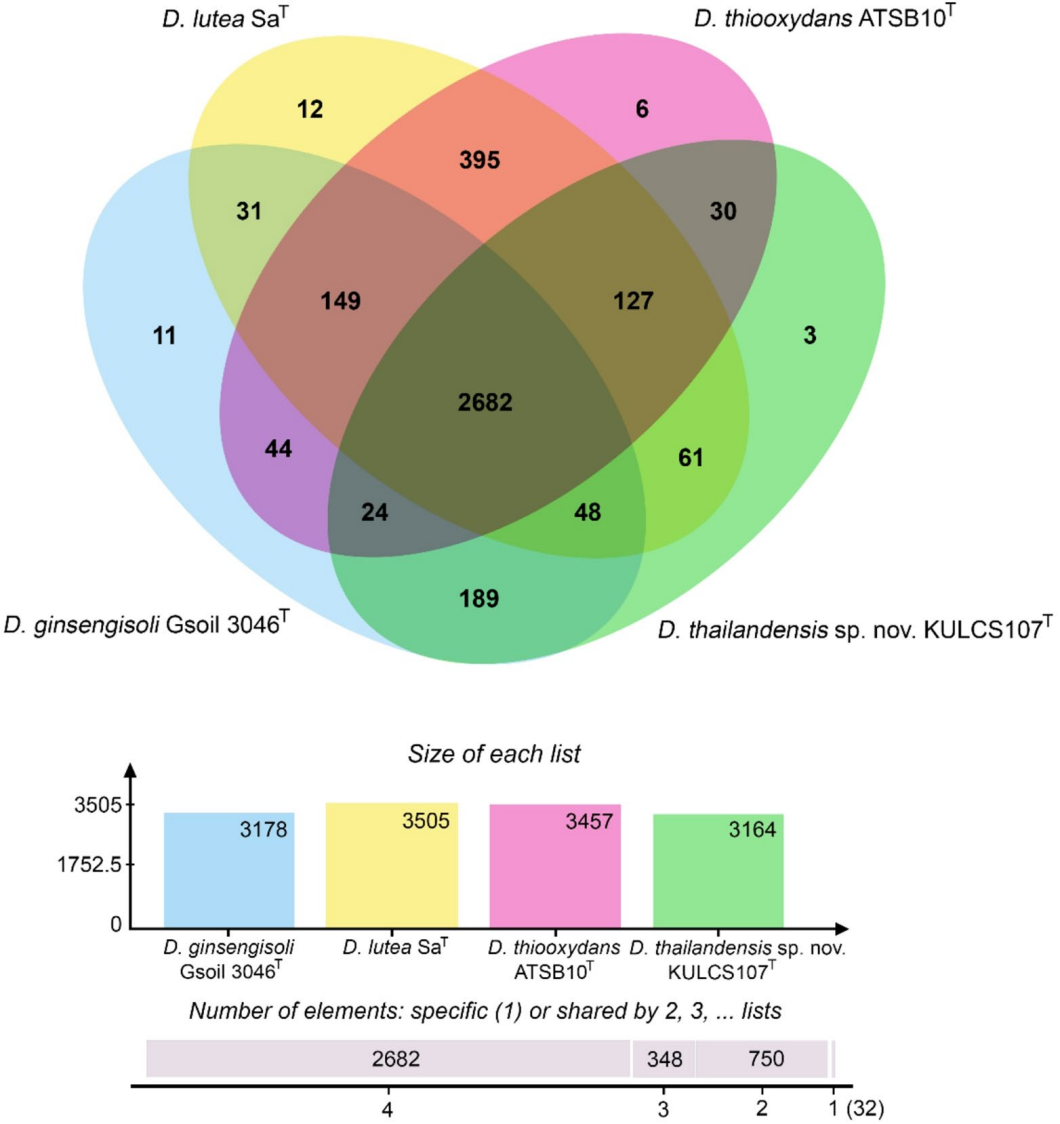
Pangenome analysis of strain KULCS107^T^ and its closest relatives. Venn diagram illustrates distribution of shared and unique orthologous gene clusters among strain KULCS107^T^ (green) and the type strains of *D. ginsengisoli* Gsoil 3046^T^ (blue), *D. lutea* SA^T^ (yellow), and *D. thiooxydans* ATSB10^T^ (pink). Bar graph indicates total number of proteins for each genome.

**Fig. 5:**
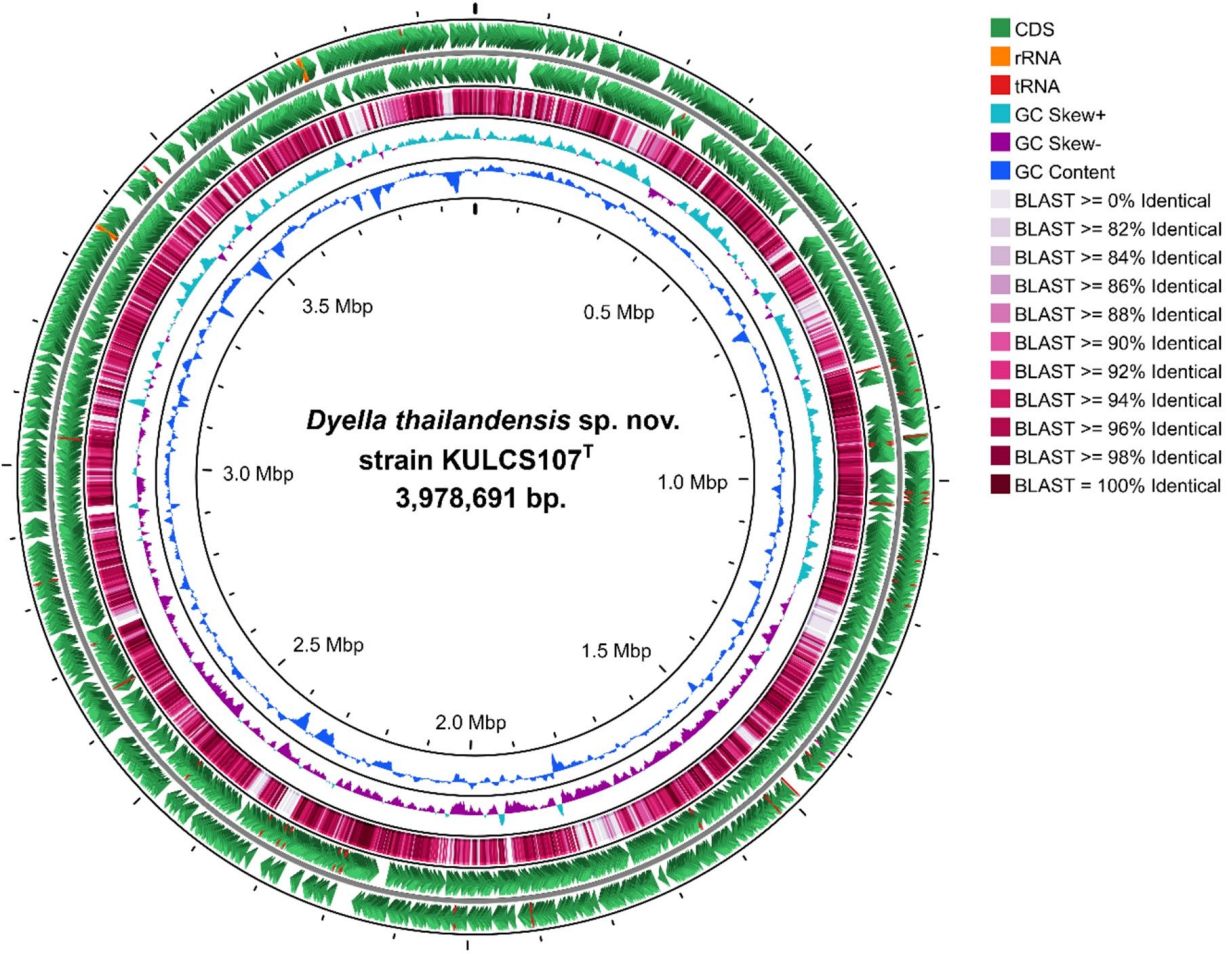
Circular genome map of strain KULCS107^T^. From outer to inner rings: protein-coding genes on forward and reverse strands (green); BLAST comparison of the CDS from KULCS107^T^ genome with *D. ginsengisoli* Gsoil 3046^T^ (color gradient from pale pink to dark red indicating level of sequence identity); GC skew (positive in cyan, negative in purple); and GC content (blue).

The phylogenetic position of strain KULCS107^T^ was strongly supported by multiple analyses. A phylogenomic tree constructed from 1,000 conserved single-copy orthologous genes showed that strain KULCS107^T^ forms a distinct and well-supported clade with *D. ginsengisoli* Gsoil 3046^T^, *D. thiooxydans* ATSB10^T^, and *D. lutea* Sa^T^, with 100% bootstrap support (Fig. 2). Furthermore, this placement was confirmed with a pairwise ANI analysis (Fig. 3). Comparative genomic analysis of KULCS107^T^ and its 3 closest relatives revealed a core genome of 2,682 orthologous gene clusters and 3 unique gene clusters in strain KULCS107^T^. Strain KULCS107^T^ shared the highest number of orthologs with *D. ginsengisoli* Gsoil 3046^T^ (2,943), further supporting their close phylogenetic proximity (Fig. 4). A circular genome map comparing KULCS107^T^ with *D. ginsengisoli* Gsoil 3046^T^ (Fig. 5) also supported this close relationship, revealing a high degree of genomic synteny and BLAST-based sequence identity. Based on this polyphasic evidence, the name *Dyella thailandensis* sp. nov. is proposed, with KULCS107^T^ designated as the type strain.

The functional capabilities of strain KULCS107^T^ and its closely related species, *D. ginsengisoli* Gsoil 3046^T^, *D. lutea* Sa^T^, and *D. thiooxydans* ATSB10^T^, were annotated and classified using the RAST subsystem (Table S1) and COG databases (Table S2). Overall, the total number of genes assigned to functional categories was slightly lower in KULCS107^T^, consistent with its smaller genome size. RAST identified 1,341 features assigned to subsystems, and COG classified 3,431 genes, both fewer than in the related genomes. All four *Dyella* strains exhibited a well-conserved core metabolic framework, with high proportions of genes associated with essential processes such as protein metabolism, amino acid and derivatives and carbohydrate (RAST), as well as transcription, amino acid transport and metabolism, and cell wall, membrane, envelope biogenesis (COG). Notably, the RAST annotation revealed marked variation in the motility and chemotaxis subsystem. Strain KULCS107^T^ encoded 62 genes in this category, a number comparable to *D. lutea* Sa^T ^(63) but nearly threefold greater than those in *D. ginsengisoli* Gsoil 3046^T^ (23) and *D. thiooxydans* ATSB10^T^ (18). This suggests that KULCS107^T^ may have a more complex chemotactic and motility system, potentially enhancing its ability to sense and respond to environmental gradients. While sharing a core metabolic framework, the genomic analysis of KULCS107^T^ reveals a distinct functional profile that, combined with its phylogenetic and genomic distinctiveness, supports its recognition as a novel species within the genus *Dyella*.

**Fig. 6:**
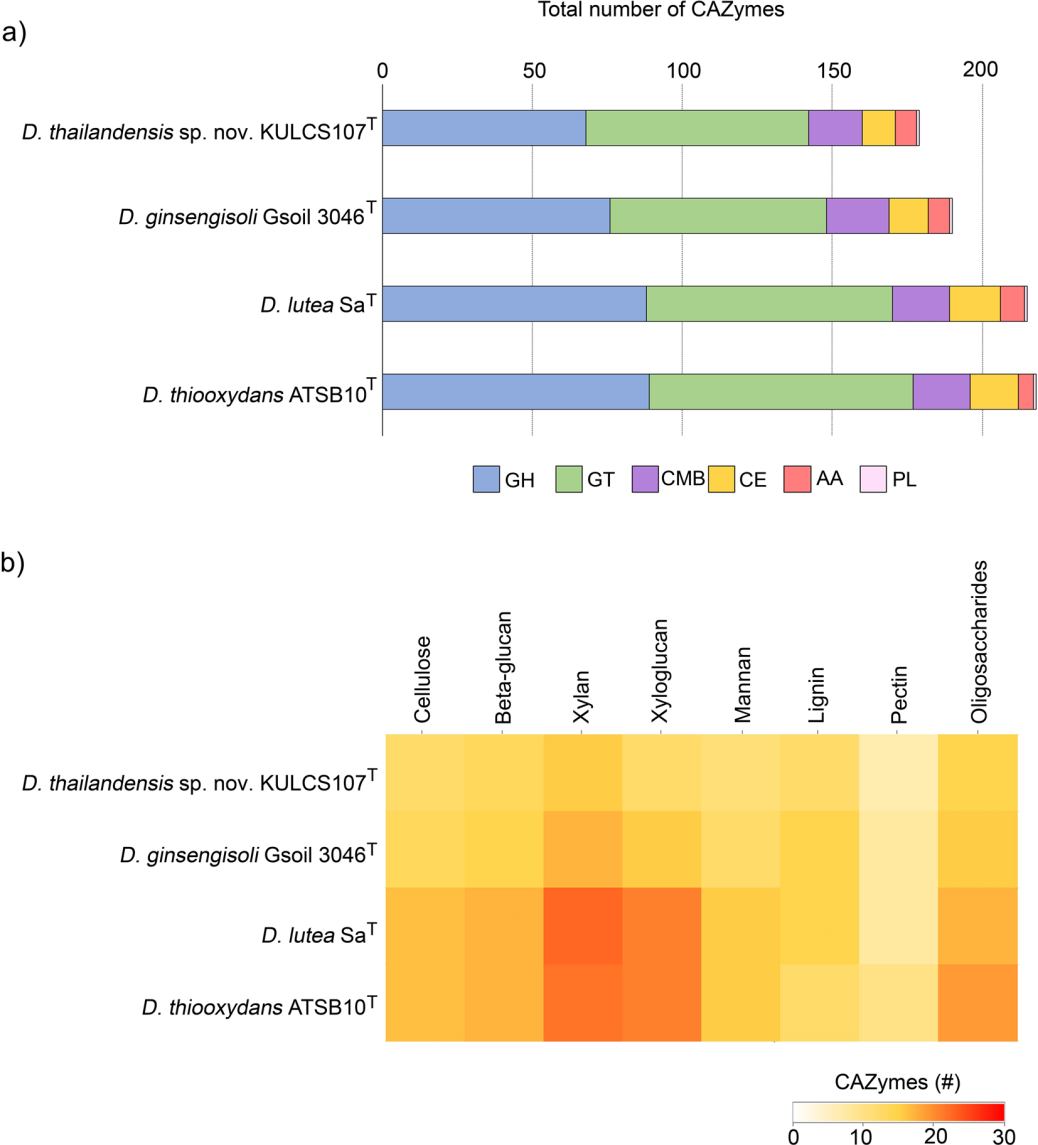
Comparative analysis of CAZyme genes in strain KULCS107^T^ and the type strains *D. ginsengisoli* Gsoil 3046^T^, *D. lutea* SA^T^, and *D. thiooxydans* ATSB10^T^. (**a**) Total number of CAZyme genes in each species. (**b**) Heatmap of CAZyme genes abundance involved in lignocellulose degradation (color gradient from light yellow to dark orange indicating gene abundance). Abbreviations: GH, glycoside hydrolases; GT, glycosyltransferases; CBM, carbohydrate-binding module; CE, carbohydrate esterase; AA, auxiliary activities; PL, polysaccharide lyase.

CAZymes are fundamental to the biovalorization of plant biomass, facilitating the degradation of complex lignocellulosic polymers such as cellulose, hemicellulose, and pectin into soluble sugars for biotechnological applications^57,58^. The genome of strain KULCS107^T^ encodes 179 putative CAZymes, a repertoire dominated by glycoside hydrolases (GHs; *n* = 68) and glycosyltransferases (GTs; *n* = 74), along with carbohydrate-binding modules (CBMs; *n* = 18), carbohydrate esterases (CEs; *n* = 11), auxiliary activities (AAs; *n* = 7), and a single polysaccharide lyase (PL) (Fig. 6a and Table S3). This repertoire is consistent with its isolation from decomposing plant material and indicates a substantial lignocellulolytic potential. Although the total number of CAZyme genes in strain KULCS107^T^ is slightly lower than in the related species *D. ginsengisoli* Gsoil 3046^T^ (190), *D. lutea* Sa^T^ (215), and *D. thiooxydans* ATSB10^T^ (218) (Fig. 6a), it remains notably higher than in several *Dyella* species isolated from the same ecological niche (subtropical forest soils), including *D. humicola* RBB1W86^T^ (119), *D. subtropica* RXD159^T^ (100), *D. silvatica* RBB189^T^ (134), and *D. silvae* RLT163^T^ (96)^6^. In addition, *Dyella jiangningensis* FCAV SCS01, recovered from the metagenome of a lignocellulose-degrading microbial consortium, contained only 36 GHs in its genome^59^. Other studies have emphasized the importance of such repertoires in other lignocellulose-degrading bacteria. For example, *Halosquirtibacter xylanolyticus* sp. nov. DS1-an-2312^T^, capable of hydrolyzing xylan into xylotriose and xylotetraose, harbors 128 GHs, 37 GTs, 27 PLs, 35 CEs, and 22 CBMs^60^. *Bacillus amyloliquefaciens*, has broad CAZyme repertoires to ferment lignocellulosic biomass, such as chrysanthemum stems, where GT and PL families are key contributors^58^. Taken together, the robust CAZyme repertoire targeting lignocellulosic substrates of strain KULCS107^T^ (Fig. 6b) positions it as a promising candidate for the deconstruction of complex plant biomass. These findings not only reinforce its ecological role in natural biomass turnover but also highlight its potential value in biomass valorization and industrial biotechnology.

### Physiological characterization

**Fig. 7:**
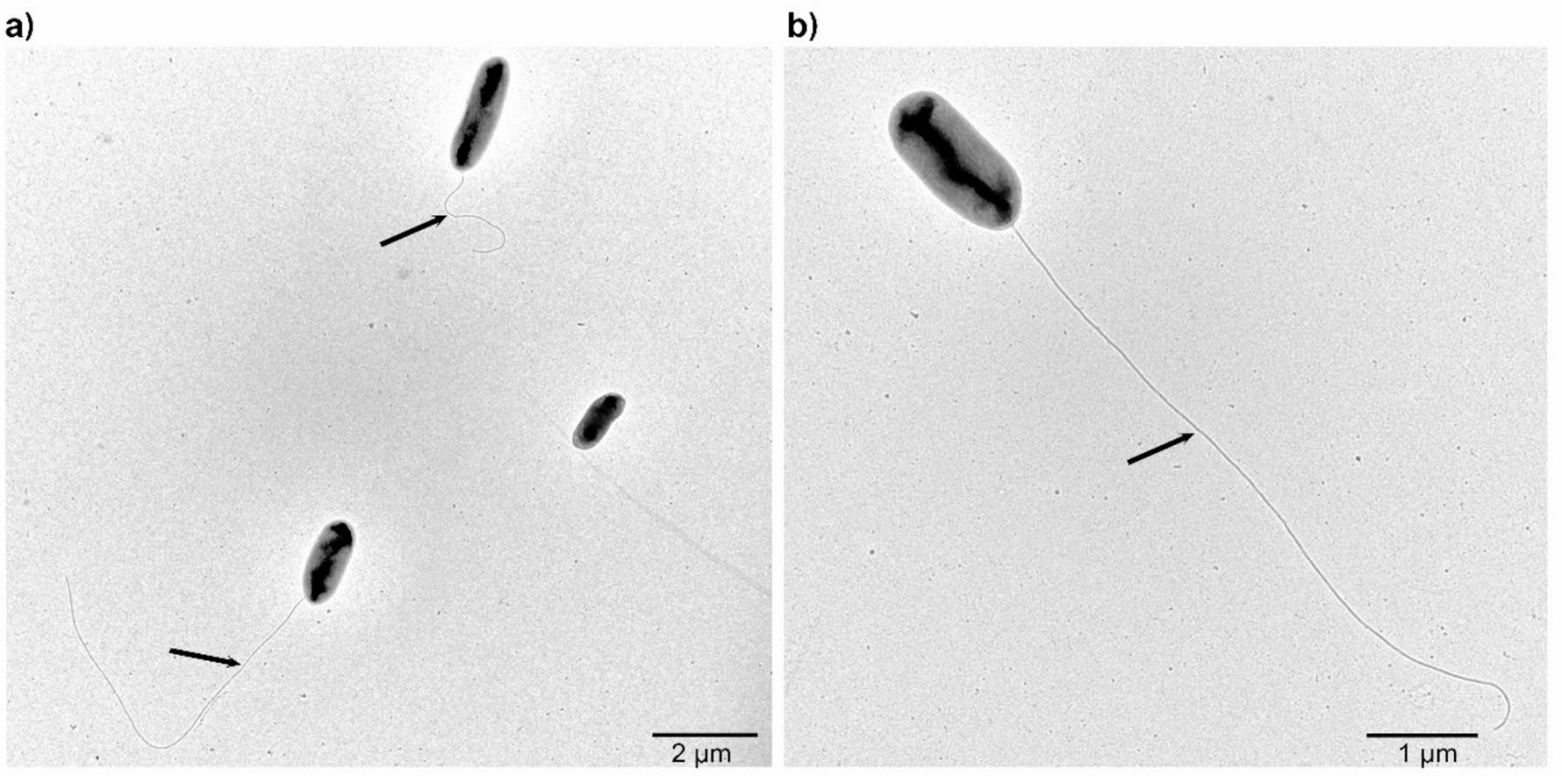
Transmission electron micrograph of cells of strain KULCS107^T^. (**a**) Cell at ×2,000 magnification. (**b**) Cell at ×4,000 magnification, where arrow indicates single polar flagellum.

Colonies of strain KULCS107^T^ were yellow-pigmented, circular, transparent, and smooth with entire margins after 2 days of incubation on R2A agar at 35 °C. Cells were Gram-negative, aerobic, rod-shaped (0.5–0.8 × 1.5–2.0 μm), and motile, with a single polar flagellum (Fig. 7). The strain was positive for both catalase and oxidase activities. Growth occurred at temperatures in the range 25–40 °C and within a pH range of 4.5–10.0. The strain tolerated NaCl concentrations up to 4.5% (w/v). A summary of detailed phenotypic characteristics is provided in Table 3. Collectively, these properties distinguish strain KULCS107^T^ from related *Dyella* species.

**Table 3:** Physiological characteristics of strain KULCS107^T^ compared with related *Dyella* species.

Characteristic	1	2	3	4	5	6
Catalase/oxidase	+/+	+/+	+/+	-/+	+/+	-/-
Temperature range (°C)	25–40	20–40	20–40	25–40	20–35	15–37
pH range	4.5–10.0	4.5–8.0	5.0–7.5	5.0–8.0	5.0–10.0	4.5–8.0
NaCl tolerance (%)	0–4.5	0–5.0	0–4.5	0–5.0	0–2.0	0–0.5
Biochemical characteristic (API 20NE)						
Nitrate reduction	-	-	-	-	-	+
Aesculin hydrolysis	+	+	+	+	-	+
Gelatinase	w	-	-	-	-	-
β-Galactosidase	-	-	-	-	-	+
Assimilation						
D-Mannose	-	-	-	w	-	-
N-Acetyl-glucosamine	+	+	w	+	w	-
D-Maltose	+	+	+	+	w	-
Enzyme activity (API ZYM)						
Alkaline phosphatase	+	+	w	+	w	-
Lipase (C14)	w	w	w	w	-	-
Cystine arylamidase	w	+	w	+	-	-
α-Chymotrypsin	w	w	w	+	-	-
Naphthol-AS-BI-phosphohydrolase	+	+	w	+	w	+
β-Galactosidase	-	-	-	-	-	+
α-Glucosidase	+	+	+	+	w	-
β-Glucosidase	+	w	+	+	-	+
N-Acetyl-β-glucosaminidase	+	+	w	+	w	-

Strains: 1, KULCS107^T^; 2, *D. ginsengisoli* Gsoil 3046^T^; 3, *D. lutea* Sa^T^; 4, *D. thiooxydans* ATSB10^T^; 5, *D. soli* JS12-10^T^; 6, *D. aluminiiresistens* A6^T^. Data for strains 1–5 obtained in the current study; data for strain 6 from Li et al.^7^. +, positive; w, weakly positive; −, negative.

### Chemotaxonomic characterization

The cellular fatty acid composition of strain KULCS107^T^ is shown in Table 4. The overall fatty acid profile of strain KULCS107^T^ was consistent with its classification within the genus *Dyella*, being dominated by branched-chain fatty acids. The major fatty acids of strain KULCS107^T^ were anteiso-C_15:0_ (18.7%), iso-C_15:0_ (13.8%), and Summed Feature 9 (13.3%). Compared to its closest relative, *D. ginsengisoli* Gsoil 3046^T^, strain KULCS107^T^ contained considerably higher proportions of anteiso-C_15:0_ (18.7% vs. 15.4%) and iso-C_15:0_ (13.8% vs. 6.8%), but lower proportions of iso-C_16:0_(8.1% vs. 15.4%) and Summed Feature 9 (13.3% vs. 16.7%). Furthermore, strain KULCS107^T^ produced a small amount of cyclo C₁₇:₀ (0.7%), which was not detected in *D. ginsengisoli* Gsoil 3046^T^. These differences provide strong chemotaxonomic support for classifying strain KULCS107^T^ as a novel species. The major respiratory quinone in strain KULCS107^T^ was ubiquinone-8, which is consistent with other members of the genus *Dyella*^7,61,62^. The major polar lipids were phosphatidylethanolamine (PE), Phosphatidylglycerol (PG), and diphosphatidylglycerol (DPG) (Fig. S5), a profile also characteristic of the genus *Dyella*^6,7,12^. The unidentified aminolipid and unidentified aminophospholipids were also present. Notably, phosphatidylserine (PS) was detected in minor amounts (Fig. S6, Table S4), which, to our knowledge, is the first report of this lipid in the genus.

**Table 4:** Cellular fatty acid composition (%) of strain KULCS107^T^compared to related*Dyella*species.

Fatty acid	1	2	3	4	5	6
Iso-C_11__:0_	6.1	4.1	3.9	3.8	3.5	3.2
Iso-C_11:0_3-OH	6.4	4.5	4.6	5.0	4.3	4.3
Iso-C_13:0_3-OH	4.1	2.3	2.4	2.5	2.4	2.9
Iso-C_14:0_	1.1	-	-	1.0	-	-
Iso-C_15:0_	**13.8**	6.8	**13.5**	**14.3**	**14.9**	7.1
Anteiso-C_15:0_	**18.7**	**15.4**	**11.41**	8.7	5.8	4.5
C_16:0_	5.5	8.5	4.1	2.7	3.3	**15.3**
Iso-C_16:0_	8.1	**15.4**	**14.4**	**18.5**	**15.1**	6.6
Iso-C_17:0_	**10.0**	**10.2**	**16.3**	**13.8**	**18.3**	**23.6**
Anteiso C_17:0_	1.7	3.2	2.3	1.7	3.5	6.4
Cyclo_17:0_	0.7	-	-	-	-	3.3
Summed Feature 3	5.3	7.4	3.8	4.1	5.4	1.2
Summed Feature 9	**13.3**	**16.7**	**17.9**	**18.0**	**16.6**	**14.3**

Major components (≥ 10.0%) are shown in bold. The symbol “–” indicates fatty acids not detected or < 1% of the total fatty acid content, except cyclo 17:0, which is reported for diagnostic significance. Strains: 1, KULCS107^T^; 2, *D. ginsengisoli* Gsoil 3046^T^; 3, *D. lutea* Sa^T^; 4, *D. thiooxydans* ATSB10^T^; 5, *D. soli* JS12-10^T^; 6, *D. aluminiiresistens* A6^T^. summed feature 3 comprises C16:1 ω7c and/or C16:1 ω6c; summed feature 9 comprises iso-C17:1 ω9c. Data for strains 1–5 were obtained in this study; data for strain 6 were from Li et al.^7^

### Lignocellulolytic enzyme activity of strain KULCS107^T^

**Fig. 8:**
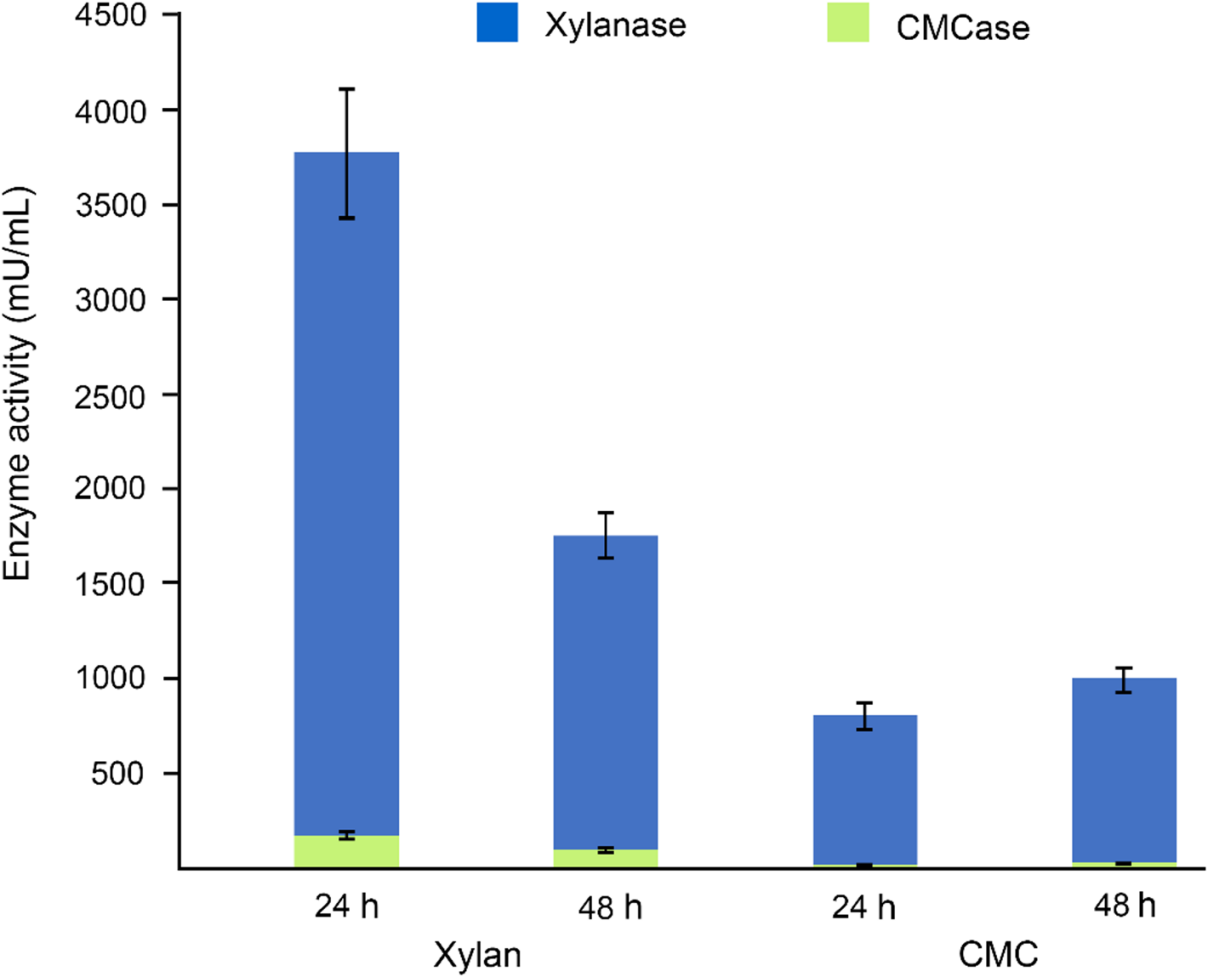
Xylanase and CMCase activities in supernatant of strain KULCS107^T^ after 24 and 48 h of cultivation in broth containing either xylan or CMC as sole carbon source.

The strain KULCS107^T^ demonstrated potent lignocellulolytic activity, with xylan induction yielding maximum xylanase and cellulase activities of 3,600 mU/mL and 180 mU/mL, respectively, at 24 h of cultivation (Fig. 8). A subsequent decline in both enzyme activities at 48 h likely resulted from nutrient depletion, catabolite repression, and enzyme degradation and deactivation^17,63^. The enzymatic profile of KULCS107^T^ was substantiated by the genomic analysis, which revealed a corresponding repertoire of CAZymes providing the genetic basis for this function (Fig. 6). The lignocellulolytic activity of KULCS107^T^ distinguishes it from its closest phylogenetic neighbors. In comparison, *D. lutea* Sa^T^ and *Dyella thiooxydans* ATSB10^T^ have no reported xylanase activity and were negative for CMC hydrolysis^61,64^, while *D. ginsengisoli* Gsoil 3046^T^ was inactive on both substrates^8^. Interestingly, these related species harbor a greater number of predicted CAZyme genes than KULCS107^T^ (Fig. 6). This discrepancy suggests that the magnitude of lignocellulolytic function in *Dyella* species is not solely determined by CAZyme gene abundance. Instead, it highlights the importance of gene expression, regulation, or differences in enzyme-specific activity. Other *Dyella* strains show variable or condition-dependent activity. For instance, *Dyella* sp. BM6 formed a hydrolysis zone on CMC agar, but its CMCase activity was not measurable in a liquid basal medium^65^. While *Dyella* sp. SSA-1562T exhibited high CMCase (6.52 U/mL) and xylanase (~ 4 U/mL) activities, those results were obtained via solid-state fermentation on sawdust^66^, limiting a direct comparison with this study.

Notably, the enzyme yields for KULCS107^T^ were achieved under unoptimized batch-culture conditions. This indicates that there is considerable potential for yield enhancement through the optimization of cultivation parameters, such as medium composition, pH, and temperature. Collectively, these findings underscore the potential of KULCS107^T^ for biotechnological applications in plant biomass deconstruction. Future studies evaluating the strain’s performance on raw, unprocessed lignocellulosic biomass are essential to fully assess its bioconversion capabilities.

### Description of *Dyella thailandensis* sp. nov.

*Dyella thailandensis* (thai.land.en′sis. N.L. fem. adj. *thailandensis* pertaining to Thailand, where the type strain was isolated). Cells are Gram-negative, aerobic, and rod-shaped (0.5–0.8 × 1.5–2.0 μm), and are motile with a single polar flagellum. Growth was observed on R2A agar at 25–40 °C (optimum, 35 °C). Growth occurred at pH 4.5–10.0 (optimum, pH 8), and in the presence of up to 4.5% (w/v) NaCl in R2A broth. The strain grows well on R2A and NA, and weakly on LB agar, but not on MacConkey agar. Catalase and oxidase are positive. In API 20NE tests, strain KULCS107^T^ hydrolyzes aesculin and shows weak gelatinase activity. Negative results are obtained for nitrate reduction, indole production, glucose acidification, arginine dihydrolase, urease, and β-galactosidase activities. The strain assimilates D-glucose, N-acetyl-glucosamine, and D-maltose, but does not utilize L-arabinose, D-mannitol, D-mannose, potassium gluconate, caprate, adipate, malate, citrate, and phenylacetate. In API ZYM tests, the strain KULCS107^T^ exhibits positive reactions for alkaline phosphatase, esterase (C4), esterase lipase (C8), leucine arylamidase, valine arylamidase, acid phosphatase, naphthol-AS-BI-phosphohydrolase, α-glucosidase, β-glucosidase, and N-acetyl-β-glucosaminidase. Weak enzymatic activities are observed for lipase (C14), cystine arylamidase, and α-chymotrypsin. Negative enzymatic activities include trypsin, α-galactosidase, β-galactosidase, β-glucuronidase, α-mannosidase, and α-fucosidase. The major fatty acids are anteiso-C_15:0_ (18.7%), iso-C_15:0_ (13.8%), and Summed Feature 9 (13.3%). The major respiratory quinone is ubiquinone-8. The major polar lipids of strain KULCS107^T^ are phosphatidylethanolamine, phosphatidylglycerol, and diphosphatidylglycerol. Phosphatidylserine is present as a minor polar lipid. The G + C content of the genomic DNA of the type strain is 67.8% based on the complete genome sequence.

## Conclusion

This study successfully characterized a novel, lignocellulolytic bacterium, strain KULCS107^T^, isolated from soil associated with leaf compost in Thailand. A polyphasic taxonomic approach, combining phylogenetic analysis of the 16S rRNA gene with genome-wide comparisons using ANI and dDDH values, demonstrated that this strain represents a novel species within the genus *Dyella*. Accordingly, the name *Dyella thailandensis* sp. nov. is proposed. The complete genome analysis revealed a relatively small genome size (3.98 Mb) with a high G + C content and an abundance of genes encoding CAZyme, consistent with its demonstrated cellulase and xylanase activities. This discovery not only contributes to the known microbial diversity but also identifies *D. thailandensis* KULCS107^T^ as a promising candidate for biotechnological applications, particularly in the biorefinery sector for the efficient conversion of lignocellulosic biomass into biofuels and other high-value biochemicals. Future studies should focus on the detailed characterization of these specific enzymes to fully harness their industrial potential.

## Supplementary Information

Below is the link to the electronic supplementary material.

Supplementary Material 1

## Data Availability

The GenBank accession numbers for the 16S rRNA gene and whole-genome sequences are PV635141 and CP192735, respectively. The SRA accession number for the PacBio (long-read) sequencing data is SRR35922960, and the Illumina (short-read) sequencing data is SRR35922961.
