# Supplementary material for: Genomic insights into a novel species, Dyella thailandensis sp. nov., a cellulolytic and xylanolytic bacterium isolated from soil associated with leaf compost
Source: Sci Rep. 2026 Jan 9;16:1287. doi: 10.1038/s41598-025-33717-w (PMC12789092; doi:10.1038/s41598-025-33717-w)
Supplement: Supplementary file 1 — Supplementary Material 1 [file 41598_2025_33717_MOESM1_ESM.docx]

**Supplementary Materials**

**Genomic Insights into a Novel Species, *Dyella thailandensis* sp. nov., a Cellulolytic and Xylanolytic Bacterium Isolated from Soil Associated with Leaf Compost**

Nahatai Intarapasit^1^, Anon Thammasittirong^1,2^, Sukanya Jeennor^3^, Pattaraporn Yukphan^4^,
Sutticha Na-Ranong Thammasittirong^1,2*^

^1^Department of Science and Bioinnovation, Faculty of Liberal Arts and Science,
Kasetsart University, Kamphaeng Saen Campus, Nakhon Pathom, 73140, Thailand,

e-mail address: nahatai.i@ku.th (Nahatai Intarapasit), anon.t@ku.ac.th (Anon Thammasittirong), sutticha.n@ku.ac.th (Sutticha Na-Ranong Thammasittirong)

^2^Microbial Biotechnology Unit, Faculty of Liberal Arts and Science, Kasetsart University,

Kamphaeng Saen Campus, Nakhon Pathom, 73140, Thailand

^3^Industrial Bioprocess Technology Research Team, Functional Ingredients and Food Innovation Research Group (IFIG), National Center for Genetic Engineering and Biotechnology (BIOTEC), National Science and Technology Development Agency (NSTDA), Pathum Thani, 12120, Thailand, e-mail address: sukanya.jee@biotec.or.th (Sukanya Jeennor)

^4^Microbial Diversity and Utilization Research Team, Thailand Bioresource Research Center (TBRC), National Center for Genetic Engineering and Biotechnology (BIOTEC), National Science and Technology Development Agency (NSTDA), Pathum Thani, 12120, Thailand, e-mail address: pattaraporn@biotec.or.th (Pattaraporn Yukphan)

*Corresponding author

Thammasittirong, S.N-R.

Tel: +663-428-1105; Fax: +663-435-1402; E-mail: sutticha.n@ku.ac.th


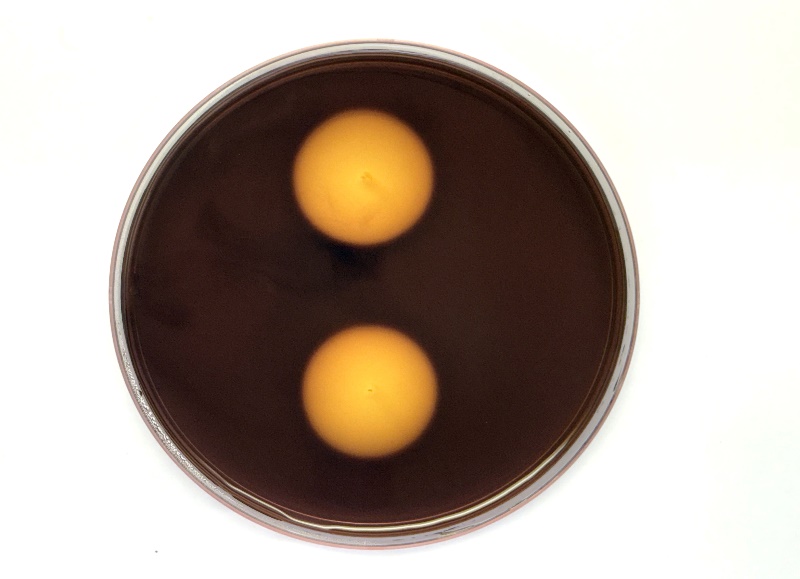

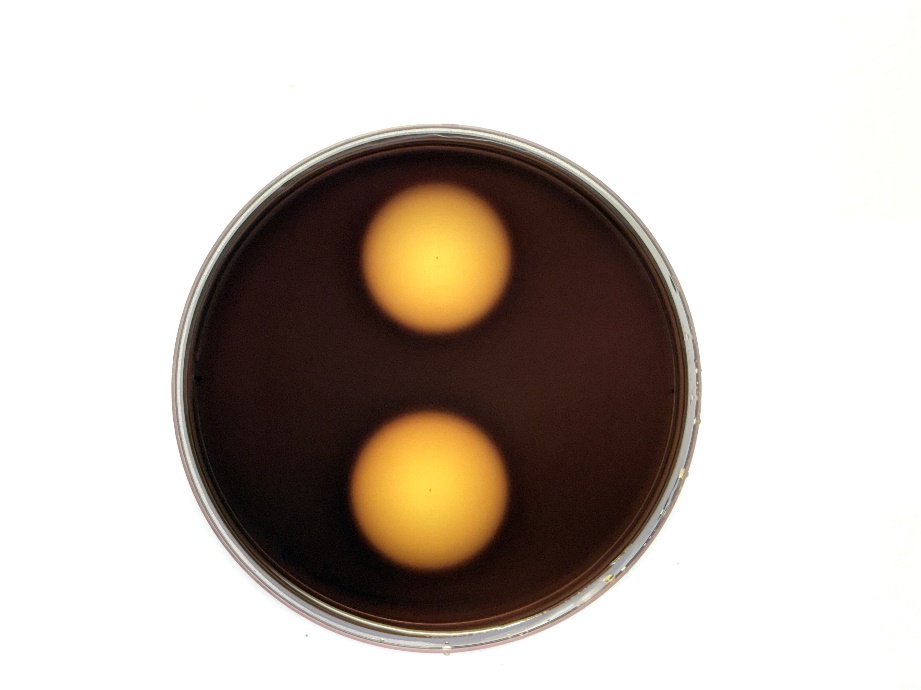


**a) b)**

**Fig. S1.** Hydrolytic activity of strain KULCS107ᵀ on different substrates. Clear zones, visualized with an iodine overlay, indicate (a) cellulase activity on CMC agar and (b) xylanase activity on xylan agar.


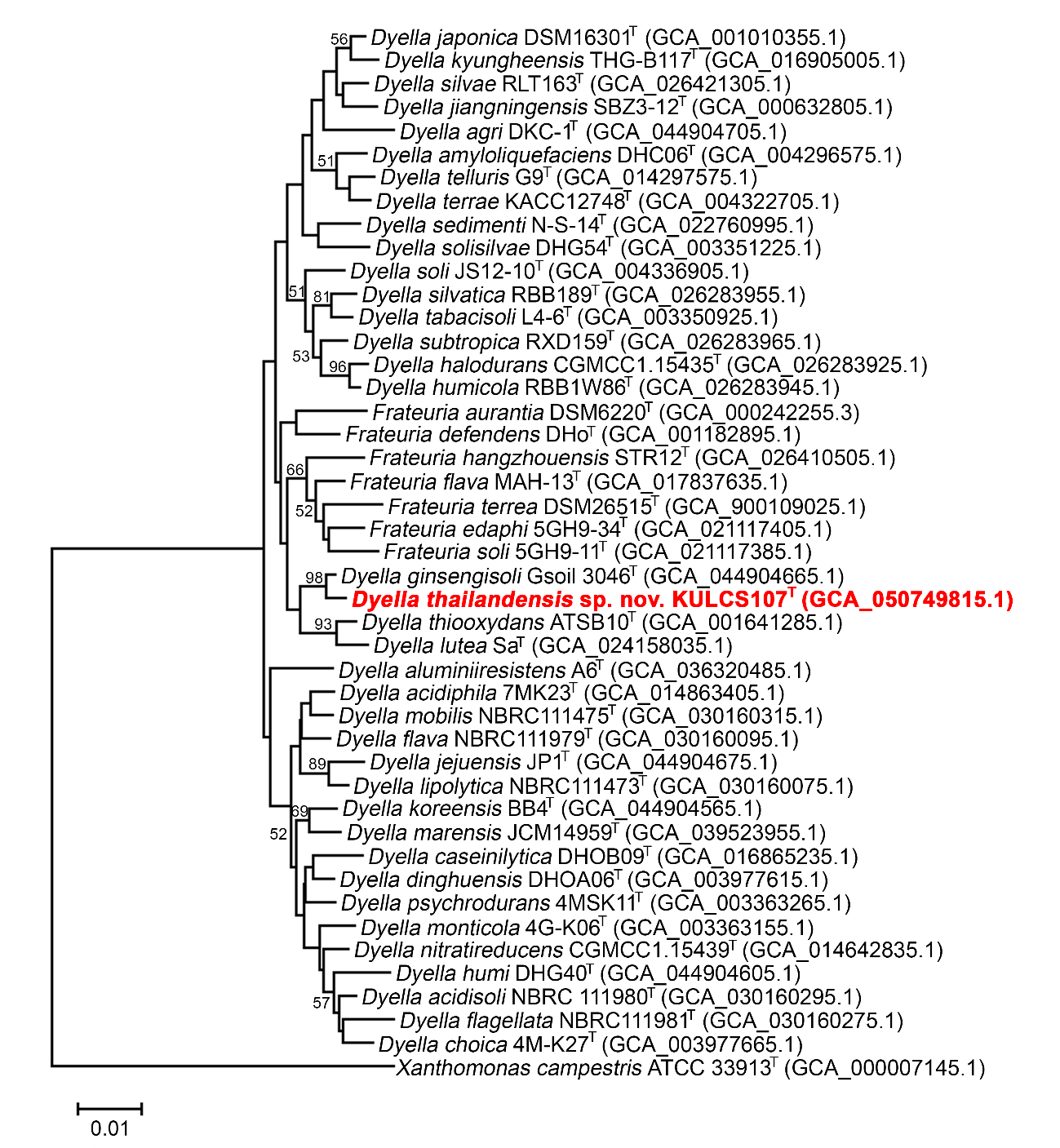


**Fig. S2** Neighbor-joining phylogenetic tree based on 16S rRNA gene sequences illustrating position of strain KULCS107ᵀ relative to all closely related species of the genera *Dyella* and *Frateuria*, with *Xanthomonas campestris* ATCC 33913ᵀ as the outgroup. Bootstrap values (based on 1,000 replications) greater than 50% are shown at the nodes. The analysis was based on an alignment of 1,545 nucleotide positions. GenBank accession numbers are in parentheses. Bar, 0.01 substitutions per nucleotide site.


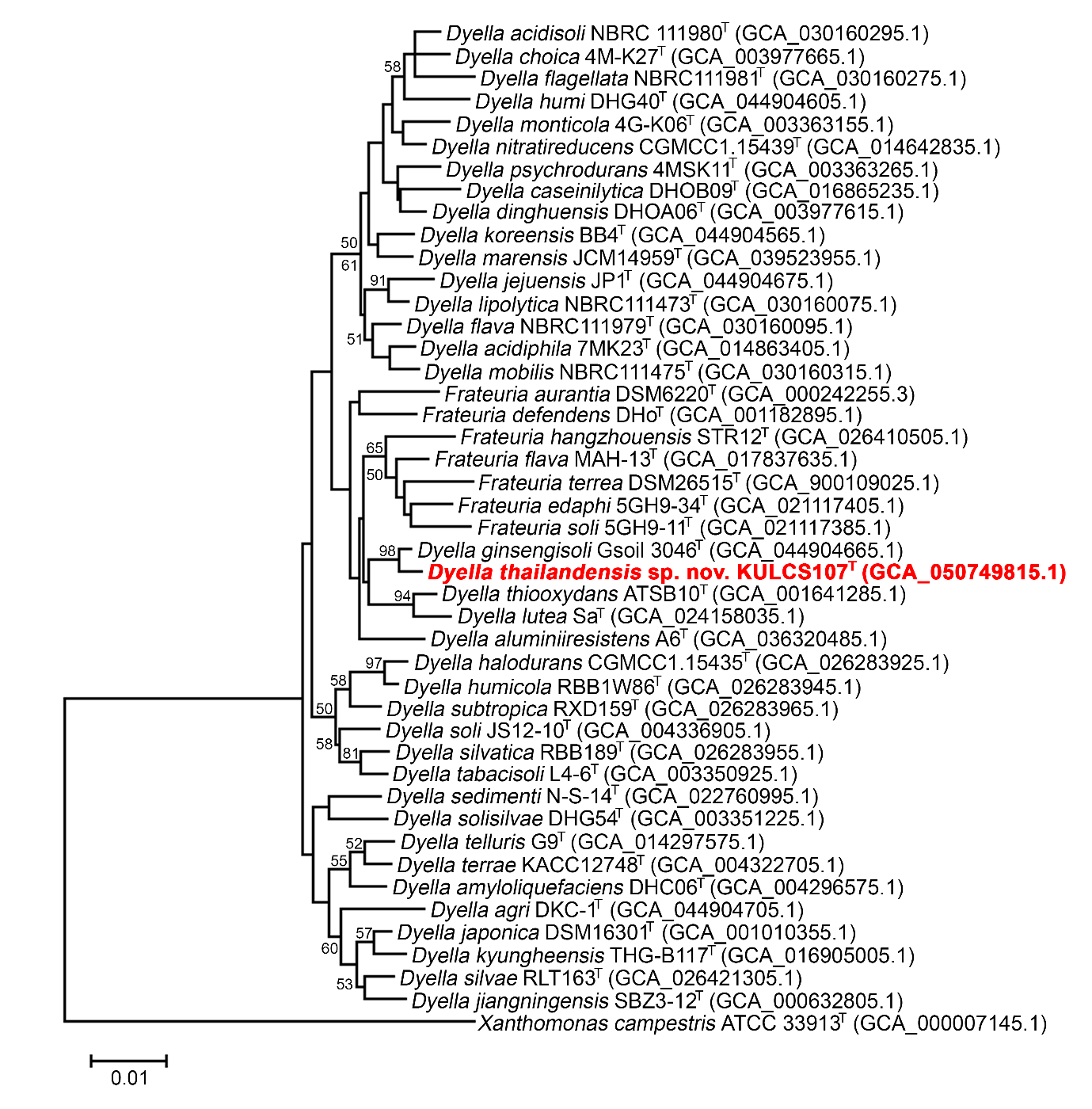


**Fig. S3** Minimum-evolution phylogenetic tree based on 16S rRNA gene sequences illustrating position of strain KULCS107ᵀ relative to all closely related species of the genera *Dyella* and *Frateuria*, with *Xanthomonas campestris* ATCC 33913ᵀ as the outgroup. Bootstrap values (based on 1,000 replications) greater than 50% are shown at the nodes. The analysis was based on an alignment of 1,545 nucleotide positions. GenBank accession numbers are in parentheses. Bar, 0.01 substitutions per nucleotide site.


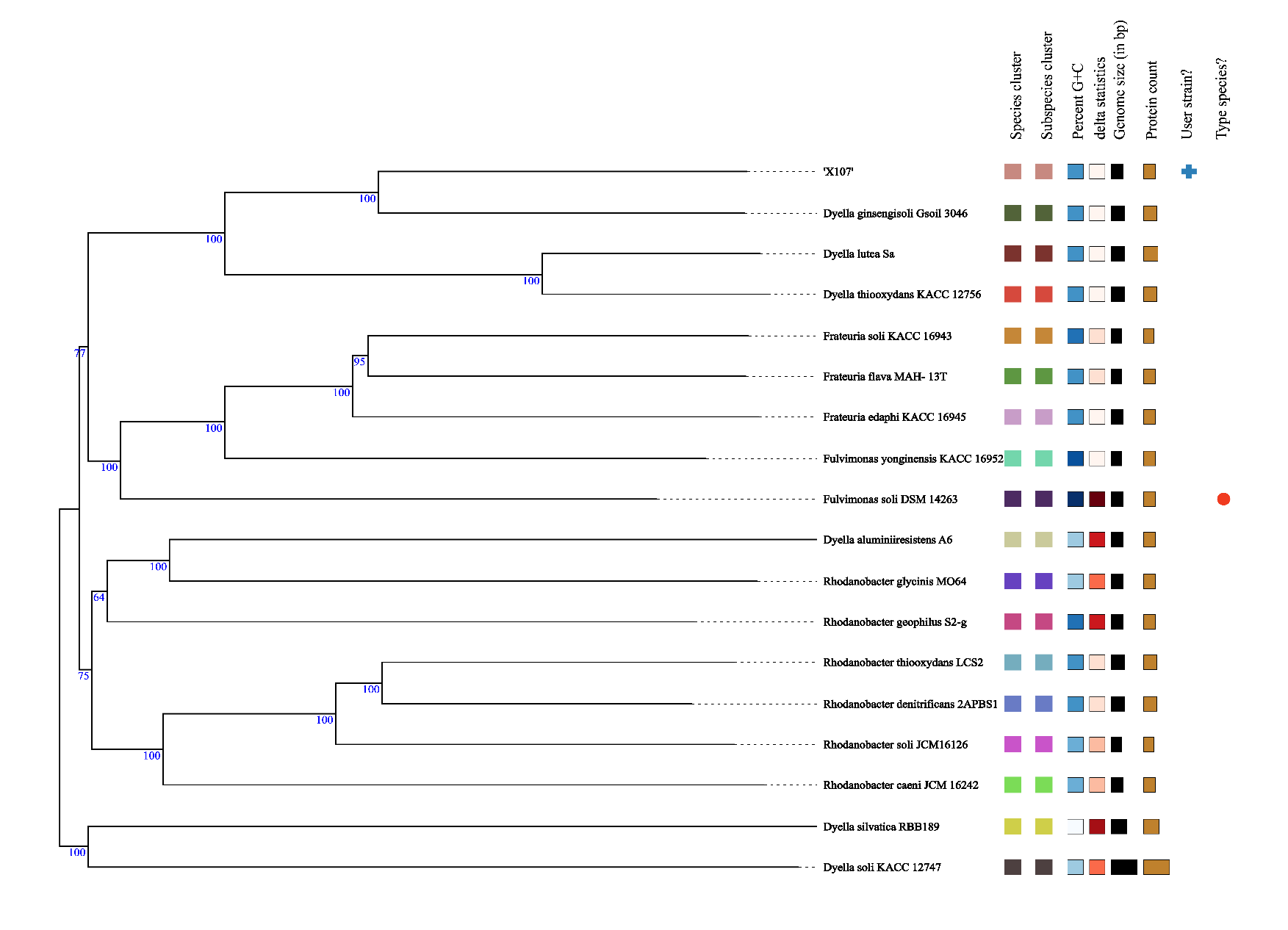


**Fig. S4.** Genome BLAST Distance Phylogeny (GBDP) tree. GBDP distances were calculated from whole-genome sequences, and the phylogenetic tree was inferred using FastME 2.1.6.1. Pseudo-bootstrap support values based on 100 replicates (average branch support: 94.1%) are shown at the corresponding nodes. The tree was rooted at the midpoint. Note: strain X107 shown in the tree corresponds to strain KULCS107ᵀ.


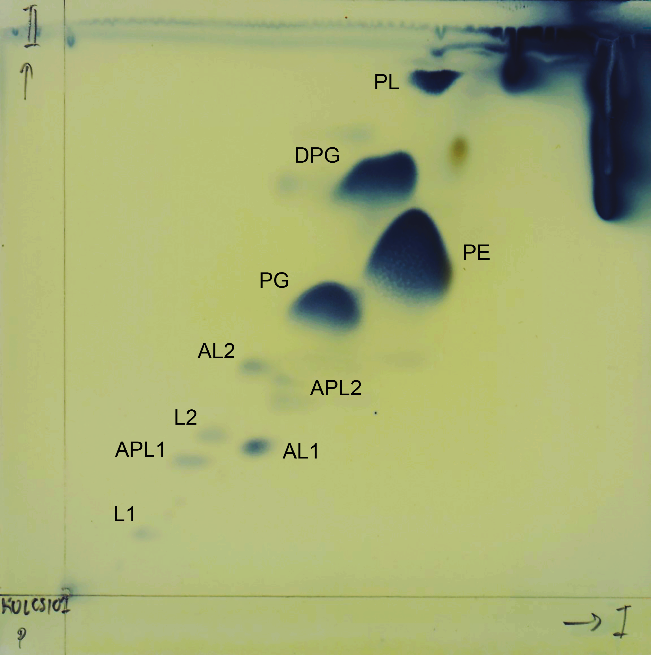


**Fig. S5** Two-dimensional TLC of the polar lipid profile from strain KULCS107ᵀ. The plate was sprayed with phosphomolybdic acid to visualize total lipids. Abbreviations: PE, phosphatidylethanolamine; PG, phosphatidylglycerol; DPG, diphosphatidylglycerol; AL, unidentified aminolipid; APL, unidentified aminophospholipid; PL, unidentified phospholipid; L, unidentified lipid.


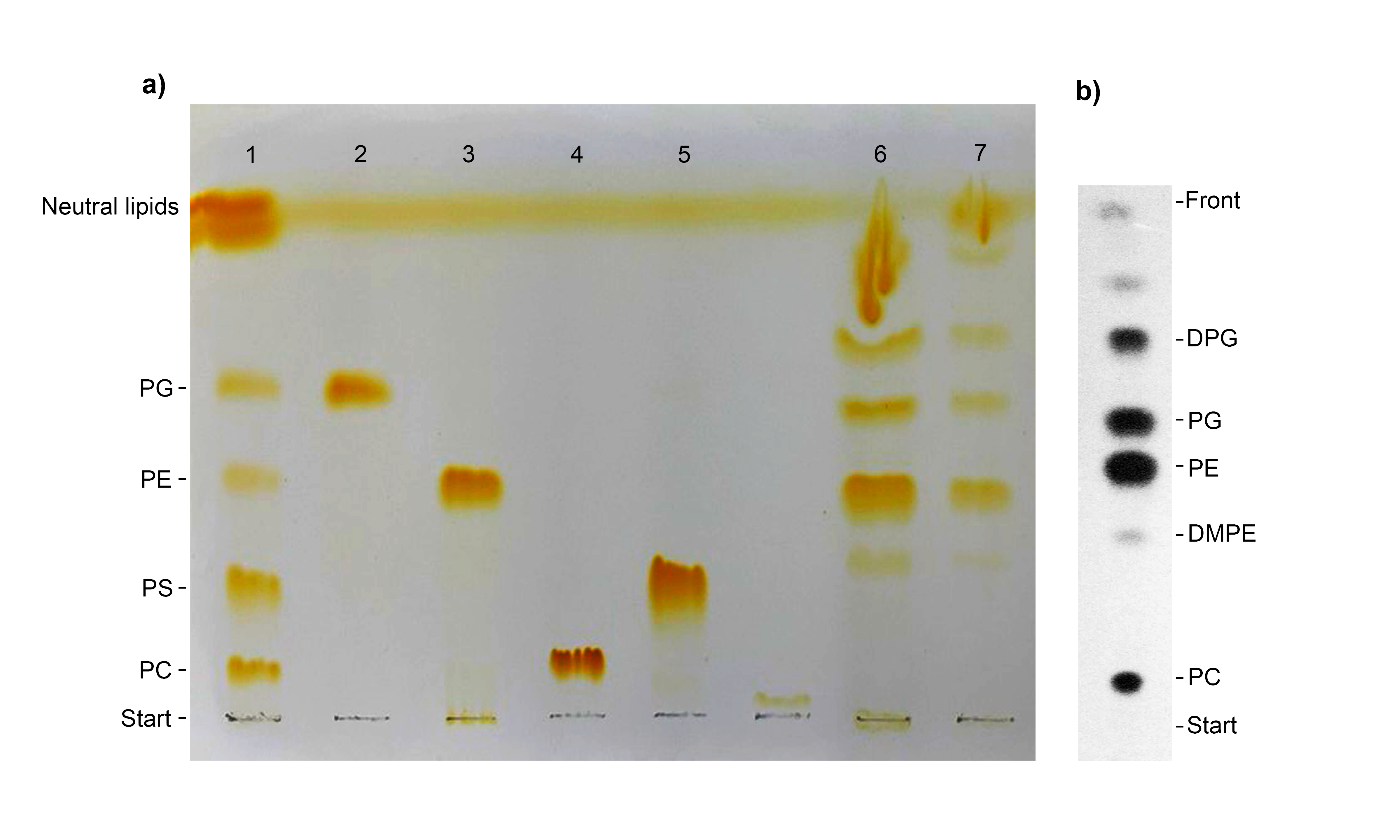


**Fig. S6** Polar lipid composition of strain KULCS107^T^ analyzed by one-dimensional HP-TLC. (a) Total lipid extract of strain KULCS107ᵀ, loaded at 0.50 mg (lane 6) and 0.25 mg (lane 7), was compared with reference standards. Lanes: 1, mixed standards; 2, PG; 3, PE; 4, PC; 5, PS. (b) Reference TLC profile of *Streptomyces coelicolor*, shown for comparison. This image is reproduced from Sandoval-Calderón et al.^50^, an open-access article distributed under the terms of the Creative Commons CC-BY license.

**Table S1.** Functional comparison of the genomes of strain KULCS107ᵀ and the type strains of *D. ginsengisoli* Gsoil 3046ᵀ, *D. lutea* SAᵀ, and *D. thiooxydans* ATSB10ᵀ based on RAST subsystem annotations. Values represent the number of gene features (count) and the percentage of the total annotated features (%) assigned to each category.

| **Subsystem feature** | **KULCS107^T^** | | ***D. ginsengisoli*  Gsoil 3046^T^** | | ***D. lutea* Sa^T^** | | | ***D. thiooxydans*  ATSB10ᵀ** | |
| --- | --- | --- | --- | --- | --- | --- | --- | --- | --- |
|  | **Count** | **%** | **Count** | **%** | **Count** | **%** | **Count** | | **%** |
| Protein metabolism | 196 | 14.62 | 201 | 14.51 | 198 | 13.61 | 195 | | 14.06 |
| Amino acids and derivatives | 189 | 14.09 | 212 | 15.31 | 192 | 13.20 | 195 | | 14.06 |
| Carbohydrates | 148 | 11.04 | 163 | 11.77 | 176 | 12.10 | 176 | | 12.69 |
| Cofactors, vitamins, prosthetic groups | 126 | 9.40 | 130 | 9.39 | 133 | 9.14 | 137 | | 9.88 |
| DNA metabolism | 83 | 6.19 | 79 | 5.70 | 70 | 4.81 | 67 | | 4.83 |
| Membrane transport | 80 | 5.97 | 84 | 6.06 | 85 | 5.84 | 87 | | 6.27 |
| Respiration | 78 | 5.82 | 80 | 5.78 | 102 | 7.01 | 102 | | 7.35 |
| Motility and chemotaxis | 62 | 4.62 | 23 | 1.66 | 63 | 4.33 | 18 | | 1.30 |
| Nucleosides and nucleotides | 61 | 4.55 | 61 | 4.40 | 58 | 3.99 | 60 | | 4.33 |
| Stress response | 61 | 4.55 | 82 | 5.92 | 79 | 5.43 | 79 | | 5.70 |
| Fatty acids, lipids, and isoprenoids | 53 | 3.95 | 53 | 3.83 | 85 | 5.84 | 61 | | 4.40 |
| RNA metabolism | 36 | 2.68 | 37 | 2.67 | 39 | 2.68 | 39 | | 2.81 |
| Phosphorus metabolism | 27 | 2.01 | 24 | 1.73 | 23 | 1.58 | 24 | | 1.73 |
| Virulence, disease and defense | 26 | 1.94 | 30 | 2.17 | 30 | 2.06 | 27 | | 1.95 |
| Cell wall and capsule | 25 | 1.86 | 28 | 2.02 | 26 | 1.79 | 25 | | 1.80 |
| Metabolism of aromatic compounds | 18 | 1.34 | 18 | 1.30 | 16 | 1.10 | 19 | | 1.37 |
| Sulfur metabolism | 16 | 1.19 | 17 | 1.23 | 15 | 1.03 | 16 | | 1.15 |
| Regulation and cell signaling | 14 | 1.04 | 11 | 0.79 | 11 | 0.76 | 7 | | 0.50 |
| Miscellaneous | 14 | 1.04 | 14 | 1.01 | 11 | 0.76 | 11 | | 0.79 |
| Nitrogen metabolism | 12 | 0.89 | 17 | 1.23 | 20 | 1.37 | 20 | | 1.44 |
| Potassium metabolism | 11 | 0.82 | 11 | 0.79 | 13 | 0.89 | 14 | | 1.01 |
| Secondary metabolism | 4 | 0.30 | 4 | 0.29 | 4 | 0.27 | 4 | | 0.29 |
| Dormancy and sporulation | 1 | 0.07 | 1 | 0.07 | 1 | 0.07 | 1 | | 0.07 |
| Phages, prophages, transposable | 0 | 0 | 5 | 0.36 | 3 | 0.21 | 3 | | 0.22 |
| Iron acquisition and metabolism | 0 | 0 | 0 | 0 | 2 | 0.14 | 0 | | 0 |
| **Total** | **1341** | **100** | **1385** | **100** | **1455** | **100** | **1387** | | **100** |

**Table S2.** Functional classification of predicted genes from strain KULCS107ᵀ and the type strains of *D. ginsengisoli* Gsoil 3046ᵀ, *D. lutea* SAᵀ, and *D. thiooxydans* ATSB10ᵀ according to COG categories. Values represent the number of genes (count) and the percentage of total genes (%) assigned to each functional category.

| **COG**  **Category** | **Description** | **KULCS107^T^** | | ***D. ginsengisoli* Gsoil** **3046^T^** | | ***D. lutea* Sa^T^** | | ***D. thiooxydans* ATSB10ᵀ** | |
| --- | --- | --- | --- | --- | --- | --- | --- | --- | --- |
|  |  | **Count** | **%** | **Count** | **%** | **Count** | **%** | **Count** | **%** |
| A | RNA processing and modification | 1 | 0.03 | 1 | 0.03 | 1 | 0.03 | 1 | 0.03 |
| B | Chromatin structure and dynamics | 1 | 0.03 | 1 | 0.03 | 1 | 0.03 | 1 | 0.03 |
| C | Energy production and conversion | 191 | 5.57 | 204 | 5.71 | 215 | 5.75 | 207 | 5.73 |
| D | Cell cycle control, cell division, chromosome partitioning | 40 | 1.17 | 40 | 1.12 | 40 | 1.07 | 40 | 1.11 |
| E | Amino acid transport and metabolism | 249 | 7.26 | 263 | 7.36 | 269 | 7.20 | 263 | 7.28 |
| F | Nucleotide transport and metabolism | 81 | 2.36 | 81 | 2.27 | 83 | 2.22 | 82 | 2.27 |
| G | Carbohydrate transport and metabolism | 152 | 4.43 | 162 | 4.53 | 180 | 4.82 | 180 | 4.98 |
| H | Coenzyme transport and metabolism | 114 | 3.32 | 118 | 3.30 | 125 | 3.34 | 128 | 3.54 |
| I | Lipid transport and metabolism | 138 | 4.02 | 140 | 3.92 | 163 | 4.36 | 160 | 4.43 |
| J | Translation, ribosomal structure and biogenesis | 192 | 5.60 | 189 | 5.29 | 195 | 5.22 | 192 | 5.31 |
| K | Transcription | 250 | 7.29 | 258 | 7.22 | 266 | 7.12 | 259 | 7.17 |
| L | Replication, recombination and repair | 149 | 4.34 | 177 | 4.95 | 149 | 3.99 | 148 | 4.10 |
| M | Cell wall, membrane, envelope biogenesis | 245 | 7.14 | 264 | 7.39 | 279 | 7.46 | 281 | 7.78 |
| N | Cell motility | 108 | 3.15 | 103 | 2.88 | 113 | 3.02 | 114 | 3.15 |
| O | Post-translational modification, protein turnover, chaperones | 146 | 4.26 | 154 | 4.31 | 165 | 4.41 | 150 | 4.15 |
| P | Inorganic ion transport and metabolism | 166 | 4.84 | 177 | 4.95 | 180 | 4.82 | 174 | 4.81 |
| Q | Secondary metabolites biosynthesis, transport and catabolism | 80 | 2.33 | 91 | 2.55 | 104 | 2.78 | 99 | 2.74 |
| S | Function unknown | 701 | 20.43 | 742 | 20.77 | 777 | 20.79 | 700 | 19.37 |
| T | Signal transduction mechanisms | 216 | 6.30 | 217 | 6.07 | 235 | 6.29 | 235 | 6.50 |
| U | Intracellular trafficking, secretion, and vesicular transport | 121 | 3.53 | 111 | 3.11 | 110 | 2.94 | 105 | 2.91 |
| V | Defense mechanisms | 85 | 2.48 | 77 | 2.16 | 85 | 2.27 | 93 | 2.57 |
| W | Extracellular structures | 2 | 0.06 | 1 | 0.03 | 1 | 0.03 | 0 | 0.00 |
| Z | Cytoskeleton | 3 | 0.09 | 2 | 0.06 | 2 | 0.05 | 2 | 0.06 |
|  | **Total** | **3431** | **100** | **3573** | **100** | **3738** | **100** | **3614** | **100** |

**Table S3.** Putative CAZyme annotations for strain KULCS107ᵀ

| **Gene ID** | **EC** | **HMMER** | **DIAMOND** | **dbCAN_sub** |
| --- | --- | --- | --- | --- |
| KULCS107_145 | 2.4.1.- | GT2(414-584)+GT2(500-697) | GH17+GT2 | GH17_e66+GT2 |
| KULCS107_1453 | - | GT2(114-338) | GT2 | GT2 |
| KULCS107_151 | - | GT51(137-307) | GT51 | GT51_e14 |
| KULCS107_1540 | 3.2.1.21\|3.2.1. | GH3(131-354) | GH3 | GH3_e1 |
| KULCS107_1553 | - | GT51(44-208) | GT51 | GT51_e71 |
| KULCS107_1689 | - | GT4(200-345) | GT4 | GT4_e1620 |
| KULCS107_1706 | - | GH23(210-332) | CBM50+GH23 | GH23_e543 |
| KULCS107_1930 | 2.4.1.182 | GT19(13-381) | GT19 | GT19_e17 |
| KULCS107_2018 | - | GT81(8-183) | GT2 | GT2 |
| KULCS107_2139 | - | GH23(246-350) | GH23 | GH23_e958 |
| KULCS107_2158 | - | GT2(8-170) | GT2 | GT2 |
| KULCS107_2159 | - | GT4(220-304) | GT4 | GT4_e1970 |
| KULCS107_2164 | - | GT4(215-364) | GT4 | GT4_e2510 |
| KULCS107_2165 | - | GT2(4-157) | GT2 | GT2 |
| KULCS107_2272 | - | GH15(226-590) | GH15 | GH15_e57 |
| KULCS107_2273 | 2.4.1.15 | GT20(6-458) | GT20 | GT20_e1 |
| KULCS107_2282 | 2.4.1.64 | GH65(348-724) | GH65 | GH65_e2 |
| KULCS107_2285 | - | GH65(361-581) | GH65 | GH65_e28 |
| KULCS107_229 | - | GH13_2(88-412) | GH13_2 | GH13_e216 |
| KULCS107_2291 | 3.2.1.\|3.2.1.113\|3.2.1.114\|3. 2.1.24 | GH92(263-753) | GH92 | GH92-e0 |
| KULCS107_2312 | 3.2.1.25 | GH2_13(38-747) | GH2_13 | GH2_e75 |
| KULCS107_2313 | 3.2.1.- | GH125(66-468) | GH125 | GH125_e1 |
| KULCS107_2314 | 3.2.1.\|3.2.1.113\|3.2.1.114\|3. 2.1.24 | GH92(296-793) | GH92 | GH92_e0 |
| KULCS107_2330 | 3.5.1.108 | CE11(4-275) | CE11 | CE11_e22 |
| KULCS107_2336 | 2.4.1.227 | GT28(185-345) | GT28 | GT28_e46 |
| KULCS107_2420 | - | GT51(58-234) | GT51 | GT51_e145 |
| KULCS107_250 | 3.2.1.20\|3.2.1.1\|3.2.1.  133 | CBM34(55-  168)+GH13_21  (228-557) | CBM34+GH13_21 | CBM34_e8+GH13_e88 |
| KULCS107_251 | - | GH31_10(228-646) | GH31_10 | GH31_e91 |
| KULCS107_252 | 3.2.1.1\|3.2.1.54 | GH13_36(44-367) | GH13_36 | GH13_e13 |
| KULCS107_2534 | - | GH43_28(56-  351)+CBM32(388-502) | CBM32+GH43_28 | GH43_e302+CBM32_e188 |
| KULCS107_256 | 3.2.1.20 | GH13_23(29-376) | GH13_23 | GH13_e140 |
| KULCS107_2581 | - | GH17(71-317) | GH17 | GH17_e51 |
| KULCS107_2582 | - | GH23(504-628) | GH23 | GH23_e550 |
| KULCS107_2584 | 3.2.1.28 | GH37(78-538) | GH37 | GH37_e7 |
| KULCS107_260 | 2.4.1.333\|2.4.1.-\|- | GH189(1009-  1552)+GH94(1778-2864) | GH189+GH94+  GT84 | GT84_e1+GH94_e9+GH94_e1 |
| KULCS107_2637 | 3.2.1.21 | GH1(7-444) | GH1 | GH1_e69 |
| KULCS107_269 | - | GH18(184-  404)+CE4(486-  590)+GT2(762-985) | CE4+GH18+GT2 | GH18_e340+CE4_e36+GT2 |
| KULCS107_2717 | - | GT2(8-121) | GT2 | GT2 |
| KULCS107_2757 | - | GT83(23-513) | GT83 | GT83_e10 |
| KULCS107_2760 | - | GT2(15-144) | GT2 | GT2 |
| KULCS107_2761 | - | GT2(36-163) | GT2 | GT2 |
| KULCS107_2762 | - | GT2(9-102) | GT2 | GT2 |
| KULCS107_2765 | - | GT83(13-503) | GT0 | GT83_e31 |
| KULCS107_2774 | 3.2.1.21\|3.2.1.74\|3.2.1.-\|3.2.1.6\|3.2.1.155 | GH3(132-354) | GH3 | GH3_e227 |
| KULCS107_2795 | 3.2.1.21\|3.2.1.74 | GH3(93-302) | GH3 | GH3_e92 |
| KULCS107_2860 | - | GT2(5-166) | GT2 | GT2 |
| KULCS107_2868 | - | GH144(42-476) | GH144 | GH144_e10 |
| KULCS107_2899 | - | GT9(102-329) | GT0 | GT9_e326 |
| KULCS107_2951 | - | GT1(8-421) | GT1 | GT1_e576 |
| KULCS107_296 | - | GH73(169-307) | GH73 | GH73_e147 |
| KULCS107_3034 | 3.2.1.28 | GH37(43-519) | GH37 | GH37_e6 |
| KULCS107_3067 | - | CE9(9-379) | CE9 | CE9_e59 |
| KULCS107_3073 | 3.2.1.52 | GH20(163-507) | GH20 | GH20_e51 |
| KULCS107_3273 | - | GT4(211-349) | GT4 | GT4_e505 |
| KULCS107_3275 | - | GT2(7-118) | GT2 | GT2 |
| KULCS107_3277 | 2.4.99.- | GT30(46-224) | GT30 | GT30_e0 |
| KULCS107_3314 | - | GH103(39-325) | GH103 | GH103_e12 |
| KULCS107_3377 | - | GH30_3(81-496) | GH30_3 | GH30_e50 |
| KULCS107_3486 | - | GT27(440-663) | GT0+GT2 | GT2+GT4_e4024 |
| KULCS107_3498 | - | GT2(6-129) | GT2 | GT2 |
| KULCS107_3502 | - | GT2(73-204) | GT2+GT4 | GT2+GT4_e3741 |
| KULCS107_3511 | - | AA3_2(2-521) | AA3_2 | AA3_e57 |
| KULCS107_3514 | - | GT2(5-167) | GT2 | GT2 |
| KULCS107_3515 | 2.4.1.129 | GT51(61-224) | GT51 | GT51_e0 |
| KULCS107_41 | - | GT2(16-235) | GT2 | GT2 |
| KULCS107_726 | - | GT9(80-307) | GT9 | GT9_e167 |
| KULCS107_83 | 3.2.1.39 | GH16_3(58-321) | GH16_3 | GH16_e303 |
| KULCS107_949 | - | GH3(54-276) | GH3 | GH3_e187 |
| KULCS107_999 | - | GH89(72-727) | GH89 | GH89_e14 |
| KULCS107_1494 | - | CE1(55-325) | N | CE1_e22 |
| KULCS107_2015 | - | AA6(5-195) | N | AA6_e2 |
| KULCS107_2091 | 1.1.3.38 | AA4(36-246) | N | AA4_e0 |
| KULCS107_2156 | N | GT105(81-239) | GT105 | N |
| KULCS107_2337 | N | GT119(26-375) | GT119 | N |
| KULCS107_2831 | - | AA1(81-585) | N | AA1_e13 |
| KULCS107_3134 | - | GH23(17-149) | N | GH23_e876 |
| KULCS107_3313 | N | GT119(48-367) | GT119 | N |
| KULCS107_357 | N | GT2(164-379) | GT0 | N |
| KULCS107_738 | - | GT83(14-369) | N | GT83_e87 |
| KULCS107_753 | - | CE14(12-141) | N | CE14_e48 |
| KULCS107_1046 | - | N | 1603 | AA2_e1 |
| KULCS107_2162 | - | N | GT4 | GT4_e3138 |
| KULCS107_2442 | - | N | CBM50 | CBM50_e555 |
| KULCS107_2758 | - | N | GT0 | GT4_e3888 |
| KULCS107_545 | - | N | GH36 | GH36_e32 |
| KULCS107_906 | - | N | CBM50 | CBM50_e36 |
| KULCS107_1537 | 3.2.1.14 | N | CBM5+GH18 | CBM5_e67+CBM5_e67+GH18_e403 |
| KULCS107_2753 | - | N | CBM50 | CBM50_e1035 |
| KULCS107_3327 | - | N | CBM50 | CBM50_e739 |
| KULCS107_917 | - | N | CBM50+GH23 | GH23_e495+CBM50_e472 |
| KULCS107_1403 | N | CBM9(36-182) | N | N |
| KULCS107_2151 | N | GT105(83-212) | N | N |
| KULCS107_2155 | N | GT105(85-223) | N | N |
| KULCS107_2175 | N | CE1(405-628) | N | N |
| KULCS107_2295 | N | GH109(15-162) | N | N |
| KULCS107_2533 | N | CE1(209-427) | N | N |
| KULCS107_2779 | N | AA1(97-353) | N | N |
| KULCS107_3023 | N | CE16(30-297) | N | N |
| KULCS107_429 | N | CE1(24-278) | N | N |
| KULCS107_633 | N | AA12(147-430) | N | N |
| KULCS107_698 | N | CE1(211-504) | N | N |
| KULCS107_3 | N | N | 118117 | N |
| KULCS107_39 | N | N | 2074615 | N |
| KULCS107_77 | N | N | CBM50+GH25 | N |
| KULCS107_82 | N | N | GH5_11 | N |
| KULCS107_92 | N | N | CE8 | N |
| KULCS107_231 | N | N | CBM35+PL11 | N |
| KULCS107_262 | N | N | CBM48+GH13_9 | N |
| KULCS107_405 | N | N | GH13_26 | N |
| KULCS107_502 | N | N | GH6 | N |
| KULCS107_546 | N | N | 372021 | N |
| KULCS107_711 | N | N | CBM50 | N |
| KULCS107_718 | N | N | GH103 | N |
| KULCS107_758 | N | N | 12577 | N |
| KULCS107_760 | N | N | 2176 | N |
| KULCS107_771 | N | N | GT2 | N |
| KULCS107_775 | N | N | GH13_48 | N |
| KULCS107_791 | N | N | GT2 | N |
| KULCS107_847 | N | N | GT1 | N |
| KULCS107_868 | N | N | GT121 | N |
| KULCS107_884 | N | N | GH0 | N |
| KULCS107_893 | N | N | 391379 | N |
| KULCS107_896 | N | N | GH13_31 | N |
| KULCS107_910 | N | N | 26967 | N |
| KULCS107_914 | N | N | 7787 | N |
| KULCS107_938 | N | N | GH28 | N |
| KULCS107_987 | N | N | GT2 | N |
| KULCS107_1075 | N | N | GT119 | N |
| KULCS107_1209 | N | N | 13268 | N |
| KULCS107_1250 | N | N | GT4 | N |
| KULCS107_1266 | N | N | 201643 | N |
| KULCS107_1287 | N | N | GH39 | N |
| KULCS107_1288 | N | N | GH39 | N |
| KULCS107_1335 | N | N | GT4 | N |
| KULCS107_1351 | N | N | CBM50 | N |
| KULCS107_1407 | N | N | GH152 | N |
| KULCS107_1442 | N | N | 20375 | N |
| KULCS107_1476 | N | N | GT4 | N |
| KULCS107_1791 | N | N | 73247 | N |
| KULCS107_1933 | N | N | GH13_30 | N |
| KULCS107_1955 | N | N | 1472 | N |
| KULCS107_1985 | N | N | 118117 | N |
| KULCS107_1988 | N | N | GH1 | N |
| KULCS107_2019 | N | N | GT1 | N |
| KULCS107_2021 | N | N | CBM5 | N |
| KULCS107_2089 | N | N | GT2 | N |
| KULCS107_2100 | N | N | GH28 | N |
| KULCS107_2161 | N | N | GT4 | N |
| KULCS107_2186 | N | N | 96008 | N |
| KULCS107_2234 | N | N | GH13_11 | N |
| KULCS107_2239 | N | N | CBM0 | N |
| KULCS107_2270 | N | N | 599368 | N |
| KULCS107_2391 | N | N | GT4 | N |
| KULCS107_2556 | N | N | GH92 | N |
| KULCS107_2564 | N | N | GH18 | N |
| KULCS107_2652 | N | N | GT30 | N |
| KULCS107_2703 | N | N | 5705 | N |
| KULCS107_2731 | N | N | GH13_8+GH13_8+842486 | N |
| KULCS107_2759 | N | N | GT121 | N |
| KULCS107_2833 | N | N | GT1 | N |
| KULCS107_2921 | N | N | GH1 | N |
| KULCS107_3047 | N | N | 5622 | N |
| KULCS107_3051 | N | N | GH0 | N |
| KULCS107_3054 | N | N | GH13_22+GT5+2304 | N |
| KULCS107_3064 | N | N | CBM50 | N |
| KULCS107_3070 | N | N | GH20 | N |
| KULCS107_3255 | N | N | GH13_23 | N |
| KULCS107_3266 | N | N | GH13 | N |
| KULCS107_3274 | N | N | GT121 | N |
| KULCS107_3312 | N | N | GH103+GT119 | N |
| KULCS107_3478 | N | N | GT4 | N |
| KULCS107_3482 | N | N | GT2 | N |
| KULCS107_3528 | N | N | GH1 | N |

**Table S4.** Polar lipid composition of strain KULCS107^T^.

^a^Identified and quantified using a commercial standard. ^b^Analyzed for using a commercial standard but was not detected. n.d., not detected. ^c^Tentatively identified based on chromatographic retention time. A standard was not available for quantification; '+' denotes presence.

| **Polar Lipid Class** | **Lipid Composition  (% of total lipids)** | **Lipid Content  (mg/g dry cell weight)** |
| --- | --- | --- |
| Phosphatidylethanolamine (PE)^a^ | 25.96 ± 3.64 | 16.40 ± 1.52 |
| Phosphatidylglycerol (PG)^a^ | 12.83 ± 1.55 | 6.49 ± 1.30 |
| Phosphatidylserine (PS)^a^ | 4.68 ± 1.34 | 1.42 ± 0.55 |
| Phosphatidylcholine (PC)^b^ | n.d. | n.d. |
| Diphosphatidylglycerol (DPG)^c^ | + | + |
